# Design of Dyes Based on the Quinoline or Quinoxaline Skeleton towards Visible Light Photoinitiators

**DOI:** 10.3390/ijms25084289

**Published:** 2024-04-12

**Authors:** Ilona Pyszka, Beata Jędrzejewska

**Affiliations:** Faculty of Chemical Technology and Engineering, Bydgoszcz University of Science and Technology, Ul. Seminaryjna 3, 85-326 Bydgoszcz, Poland; beata@pbs.edu.pl

**Keywords:** photopolymerization, UV-Vis initiators, quinoline derivatives, quinoxaline derivatives, two-component initiating systems

## Abstract

Dyes based on quinoline and quinoxaline skeletons were designed for application as visible light photoinitiators. The obtained compounds absorb electromagnetic radiation on the border between ultraviolet and visible light, which allows the use of dental lamps as light sources during the initiation of the photopolymerization reaction. Their another desirable feature is the ability to create a long-lived excited state, which enables the chain reaction to proceed through the mechanism of intermolecular electron transfer. In two-component photoinitiating systems, in the presence of an electron donor or a hydrogen atom donor, the synthesized compounds show excellent abilities to photoinitiate the polymerization of acrylates. In control tests, the efficiency of photopolymerization using modified quinoline and quinoxaline derivatives is comparable to that obtained using a typical, commercial photoinitiator for dentistry, camphorquinone. Moreover, the use of the tested compounds requires a small amount of photoinitiator (only 0.04% by weight) to initiate the reaction. The research also showed a significant acceleration of the photopolymerization process and shortening of the reaction time. In practice, this means that the new two-component initiating systems can be used in much lower concentrations without slowing down the speed of obtaining polymer materials. It is worth emphasizing that these two features of the new initiating system allow for cost reduction by reducing financial outlays on both materials (photoinitiators) and electricity.

## 1. Introduction

A huge problem in the modern world is global climate change, caused mainly by carbon dioxide emissions. It is related to the processes of burning fossil fuels in electricity generation and heat production, transportation and industry. The energy sector is the source of about three-quarters of global greenhouse gas emissions [1]. Switching to renewable energy sources such as solar, wind and hydropower and improving energy efficiency are essential to save the environment. Nowadays, increased attention is paid to the amount of energy used in chemical reactions. Therefore, it is increasingly proposed to apply light as an energy source [2]. Most photochemical-based manufacturing processes can effectively avoid high energy consumption, which is crucial in practical industrial production.

In recent years, the most innovative technologies for producing polymeric materials are based on processes initiated photochemically, i.e., with light [3,4,5,6,7,8]. This technique is quite common nowadays. The range of applications of photopolymerization is also confirmed by the growing interest in photoinitiating systems operating in the visible light range. It is worth noting that the most popular applications of photopolymerization include dentistry, 3D/4D printing and coating [9,10,11,12,13,14,15,16,17,18,19]. Moreover, the synthesis of polymeric materials conducted by photopolymerization is one of the most efficient methods. Due to the low energy consumption, lack of solvents and the high speed of the process at ambient temperature, photopolymerization is also an environmentally friendly technique [10]. The growing interest in photopolymerization processes leads to research aimed at increasing the efficiency of the process of obtaining polymers using visible light. This is mainly because visible light, compared to ultraviolet light, is cheaper, safer and has a greater ability to penetrate deep into the polymerizing mixture [20].

However, commercial initiators used in industry—compounds responsible for starting photopolymerization processes—have significant limitations. The photoinitiators used so far absorb visible light to a small extent. This is an extremely important technological problem for their versatile applications because these compounds absorb radiation emitted by cheap light sources in an ineffective way.

Photoinitiators, next to co-initiators, monomers and additives responsible for the specific functional requirements of the materials, are the main components of photopolymerization systems. The photoinitiator plays a key role in the photoinitiation system and therefore in the entire photopolymerization process, because it directly determines the photopolymerization rate and also affects the physical and mechanical properties of the polymerized materials [21]. According to the type of photoinitiator used, the photopolymerization reaction can be divided into free radical photopolymerization, cationic photopolymerization and anionic photopolymerization [22]. In the case of the former, photoinitiators must have the ability to absorb light in the appropriate spectral range (UV or Vis) [23,24]. When exposed to UV or visible light, photoinitiators absorb photons and then undergo subsequent reactions out of the excited state, creating free radicals capable of inducing a chain reaction. When multifunctional monomers are used, cross-linked structures are created [25].

Photoinitiators of free radical polymerization, depending on the mechanism of radical formation, are divided into two main groups, i.e., type I and type II [26,27,28]. Type I photoinitiators are directly cleaved upon irradiation to generate initiating radicals, while type II photoinitiators generate radicals by photoinduced electron transfer (PET) between the photoinitiator and co-initiator [29,30,31,32]. Type II photoinitiators are aromatic ketones, e.g., benzophenone [33], as well as cyanines [34], phenazines [35] and many others [32,36,37,38,39,40]. Among them, there are those that have good light absorption properties and high initiation efficiency in the UV region, which makes them extremely popular photoinitiators in the field of UV curing.

The co-initiators are usually electron donors, i.e., aromatic amines or sulfur compounds [24,41,42]. However, amine additives are often toxic [43]. Due to this serious drawback, new co-initiators with low toxicity are being sought.

Owing to certain inconveniences associated with the use of commercial photoinitiators, there was a need to develop new, universal and effective photoinitiator systems that would be more sensitive to visible light sources. Therefore, an attempt was made to obtain effective photoinitiators by modifying the structure of dyes containing quinoline and quinoxaline skeletons to improve the photoinitiating properties in the visible region. The aim of the research was also to significantly accelerate the process, shorten the polymerization time and reduce the concentration of the initiating system and, thus, reduce costs resulting from lower financial outlays on both materials (photoinitiators) and electricity. In practice, this means that new two-component initiating systems can be used in much lower concentrations without slowing down the speed of obtaining polymer materials. The commercial attractiveness of the designed initiating systems results from the possibility of their use in the dental industry for obtaining light-cured dental fillings, in medicine for obtaining hydrogel polymeric materials, as well as in modern imaging techniques using 3D printing.

## 2. Results and Discussion

### 2.1. Designing the Structure of the Dyes

Five compounds containing a quinoline (DQ1, DQ2) or quinoxaline (DQ3, DQ4, DQ5) skeleton were synthesized. The selection of compounds containing a nitrogen-based heterocyclic ring allows with high probability to design compounds with biological, chemotherapeutic and pharmacological applications, such as antirheumatic and antihistamine drugs [44,45]. Moreover, 2-aminopyridine, quinoline and quinoxaline have anticancer, antibacterial, antiviral, antimalarial and antifungal properties [46,47,48,49,50]. This is beneficial from the viewpoint of further potential use of these dyes as photoinitiators in dentistry. In addition, their structure was stiffened by condensation of several aromatic rings. This was intended to eliminate excited state deactivation channels by preventing the rotation of benzene rings and isomerization.

The criterion for selecting dyes was not only the ease of synthesis of these compounds, but also their spectroscopic properties. Therefore, the structure of the quinoline derivative DQ2 was modified to the quinoxaline derivative (DQ3, DQ4) to adjust the spectroscopic properties, especially the position and intensity of the absorption band, and to obtain compounds that effectively initiate polymerization upon visible light irradiation. The synthesized derivatives differ in the number of nitrogen atoms (DQ1–DQ5), the presence of a methyl substituent (DQ4), or an oxygen atom (DQ5).

The structure and purity of the dyes were confirmed by NMR spectroscopy. The lack of a signal at 3.0–5.0 ppm in the ^1^H NMR spectra, indicating the presence of an amino group, confirmed the formation of a condensation product with a characteristic –C=N– bond (signal at 150.0–160.0 ppm in the ^13^C NMR spectrum). All synthesized compounds (DQ1–DQ5) were in the form of light-yellow crystals, and their solutions were pale yellow in color. This is especially important from the viewpoint of their potential use in dentistry because it allows for obtaining natural shades of fillings.

### 2.2. Spectroscopic Properties

The electronic absorption spectra of the synthesized dyes were recorded in ethyl acetate and are presented in Figure S1 in Supplementary Materials. On their basis, both the wavelength at which the absorption maximum occurs (*λ_max_*) and the molar absorption coefficients (*ε*) were determined (Table 1).

Unsubstituted quinoline (QL) and quinoxaline (QX) absorb electromagnetic radiation in the UV region. The absorption maxima of these compounds are at 204, 225 and 276 nm for QL [45] and at 314 nm for QX [51]. The data collected in Table 1 and shown in Figure S1 indicate that the modification of these structures clearly affects the position of the absorption bands and induces the bathochromic effect. DQ2 and DQ3 dyes absorb electromagnetic radiation on the border between ultraviolet and visible light region. However, the absorption maxima of DQ1 and DQ4 dyes are in the visible range, or, as in the case of DQ5, the absorption bands are wide and overlap the visible region. Thanks to this, they can absorb light emitted by the dental lamp used in the photopolymerization process. Moreover, their molar absorption coefficients at 400 nm range from 3000 M^−1^ cm^−1^ to 4000 M^−1^ cm^−1^ and are approximately 500 times higher than those of camphorquinone (8 M^−1^ cm^−1^ at 400 nm), a standard photoinitiator used in dentistry.

Furthermore, the modification of the quinoline skeleton (DQ2) to 2-oxo-2,3-dihydro-1*H*-imidazo[1,2-a]pyridinium bromide (DQ1) (Figure S1) causes a change in the shape of the absorption band and shifts its maximum to a long wavelength by approximately 70 nm (bathochromic effect). The DQ1 dye has an electronic absorption spectrum characterized by several maxima in the long-wavelength part of the spectrum. This is typical for heterocyclic aromatic compounds with fused rings. However, the modification of the quinoline ring (DQ2) to quinoxaline (DQ3 and DQ4) does not change the shape of the absorption bands but only shifts them towards longer wavelengths by 18–38 nm (Figure S1). The electronic absorption spectra of these dyes are characterized by an intense absorption band located in the range of 320–350 nm and a second, less intense absorption band shifted by ca. 35–50 nm to the red. The presence of an oxygen atom in quinoxaline derivatives (DQ5) compared to the quinoline derivative (DQ2) causes a decrease in the intensity of the long-wavelength absorption band with a simultaneous bathochromic effect (Figure S1).

After absorbing electromagnetic radiation, the dye molecule returns to its ground state, emitting excess energy as electromagnetic radiation or heat. The emission spectra provide valuable information about the excited states of the molecule and the mechanism of energy transfer between them. The phenomenon of photoluminescence, both fluorescence and phosphorescence, was observed for the synthesized dyes.

Similarly to absorption spectra, the wavelength at which the fluorescence and phosphorescence maximum occurs depends on the structure of the synthesized dyes. The data presented in Figure S2a,b indicate the bathochromic effect observed because of the modification of the structure of the DQ2 dye. Compound DQ1 has complex fluorescence with strong peaks occurring at 447, 477 and 511 nm. The remaining DQ2-DQ5 compounds have broad fluorescence bands with one maximum, shifting towards longer wavelengths with the increase in the number of nitrogen atoms in the molecule (DQ2 vs. DQ1, DQ3, DQ4) and the introduction of an oxygen atom (DQ2 vs. DQ5). This means that the introduction of additional nitrogen atoms in the dye molecule, and thus the modification of quinoline derivatives to quinoxaline, is responsible for shifting the fluorescence band to the longer wavelength (lower frequency).

**Figure 1:**
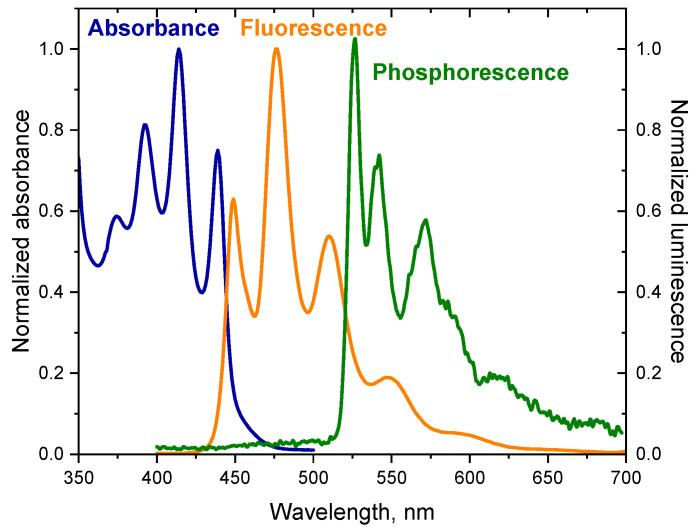
Electronic absorption spectrum, fluorescence and phosphorescence spectra of 2-oxo-2,3-dihydro-1*H*-imidazo[1,2-a]pyridinium bromide (DQ1) in 2-methyltetrahydrofuran. The phosphorescence spectrum was recorded at liquid nitrogen temperature; Ex = 350 nm.

In addition to fluorescence, the dyes tested also emit phosphorescence. The phosphorescence spectrum has a longer wavelength than the fluorescence spectrum because the energy of the triplet state is lower than the energy of the corresponding singlet state [49]. The spectra presented in Figure 1 illustrate the relationship between the absorption spectrum and emission spectra of the dye (DQ1) (the spectra for other dyes are included in Supplementary Materials—Figure S3) [52]. The energy values of the T_1_ → S_0_ transition ($E_{T}^{00}$) were determined based on the phosphorescence spectra. The energy of the triplet state for the synthesized dyes ranges from 2.51 to 2.95 eV. The spectroscopic data are collected in Table 1.

Moreover, the fluorescence spectra of the synthesized dyes are a beautiful illustration of the principle of mirror image electronic absorption spectra (Figure 1).

**Figure 2:**
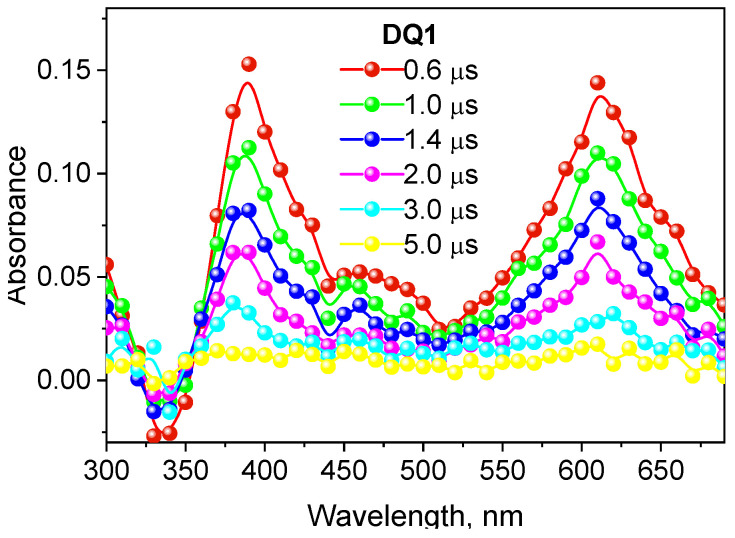
Absorption spectrum of the triplet state of 2-oxo-2,3-dihydro-1*H*-imidazo[1,2-a]pyridinium bromide (DQ1) recorded in deoxygenated acetonitrile.

**Figure 3:**
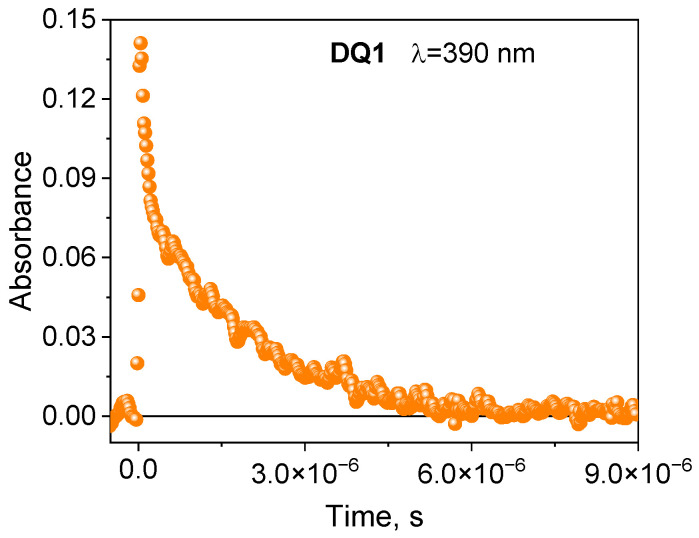
Kinetic curves of the disappearance of the triplet state recorded for 2-oxo-2,3-dihydro-1*H*-imidazo[1,2-a]pyridinium bromide (DQ1) at 390 nm.

The formation of the triplet state by the tested dyes was also confirmed by recording transient absorption spectra using the laser flash photolysis method. The spectral characteristics of the DQ1-DQ5 triplet state are shown in Figure 2 and Figure S4 in the Supplementary Materials. After excitation of the dye with a laser light beam at 355 nm in a deoxygenated acetonitrile, both quinoline and quinoxaline derivatives show transient absorption that decays on a microsecond time scale. The absorption bands at 340–430 nm are attributed to triplet state absorption. The maxima of these bands depend on the dye structure, and are located at 390 nm, 430 nm, 370 nm, 360 nm and 340 nm for DQ1, DQ2, DQ3, DQ4 and DQ5, respectively. The analysis of the transient absorption kinetic curves (Figure 3 and Figure S5 in Supplementary Materials) allowed to determine the lifetime of the triplet state. Its value is 1.52, 0.74, 1.31, 1.58 and 1.00 μs for DQ1, DQ2, DQ3, DQ4 and DQ5, respectively. It can be concluded that the lifetime of the triplet state of the DQ1 and DQ4 derivatives is approximately twice as long as that of the DQ2 derivative. The DQ1 and DQ4 derivatives also show higher intersystem transition efficiency compared to DQ2 and DQ3, as evidenced by the triplet state quantum yield values of 0.549, 0.006, 0.106, 0.632 and 0.270 for DQ1, DQ2, DQ3, DQ4 and DQ5, respectively (Table 2). The quantum yield of the triplet state was determined using the method described by Lament et al. [53] and by us [35].

The negative absorption with maximum at 390 nm, 430 nm, 370 nm, 360 nm and 340 nm for DQ1, DQ2, DQ3, DQ4 and DQ5, respectively, is due to photophysical processes and corresponds to the high ground-state absorption at these wavelengths (Figure 2 and Figure S4).

**Figure 4:**
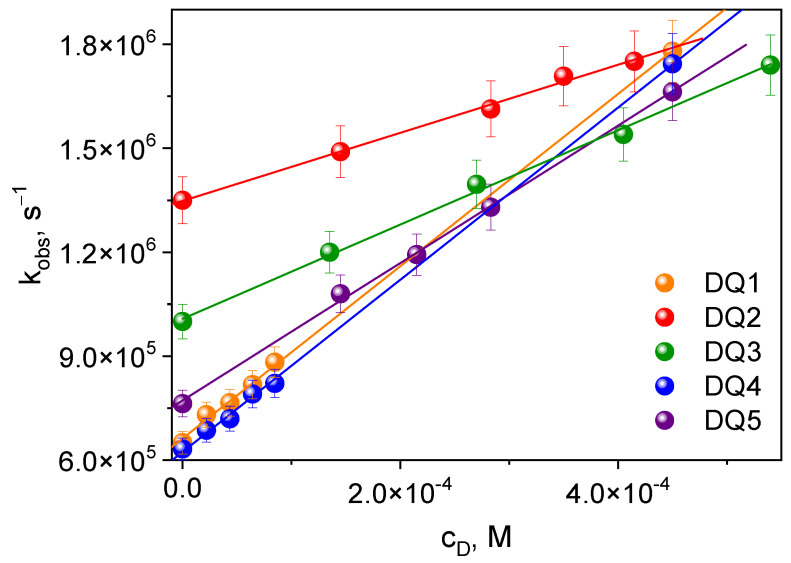
Stern–Volmer dependence of the triplet state quenching process for the tested dyes. Electron donor: thiophenoxyacetic acid.

The transient absorption spectra confirm that these compounds generate a triplet state. This is particularly important in the context of initiating the polymerization reaction. The triplet state of the synthesized dyes was effectively quenched by electron donors used in the tested photoinitiating systems: PhTAA, PhTACA, DMA, PhIAA, PhAA and PhPA. The quenching rate constants *k_q_* (Table 3) were determined from the triplet–triplet transient absorption spectra at a specific wavelength, recorded at different quencher concentrations based on the classical Stern–Volmer equation [54] (Figure 4):

*k_obs_* = τ_T_^−1^ + *k_q_*[Q] (1)

where: *k_q_*—quenching rate constant of the excited state, τ_T_—lifetime of the excited state in the absence of an electron donor, [Q]—molar concentration of the quencher.

If the only way to quench the triplet state is by the electron donor, it can be assumed that the quenching rate constant is equal to the electron transfer rate constant [27].

*k_q_* = *k_el_* (2)

The research shows that the rate of quenching of the excited state depends on the structure of the dye. The triplet state of DQ1 and DQ4 dyes is effectively quenched by thiophenoxyacetic acid and other electron donors used; therefore, higher electron transfer rate constants are observed for them compared to the other dyes. Consequently, they should better initiate the polymerization of triacrylates.

### 2.3. Photopolymerization

**Figure 5:**
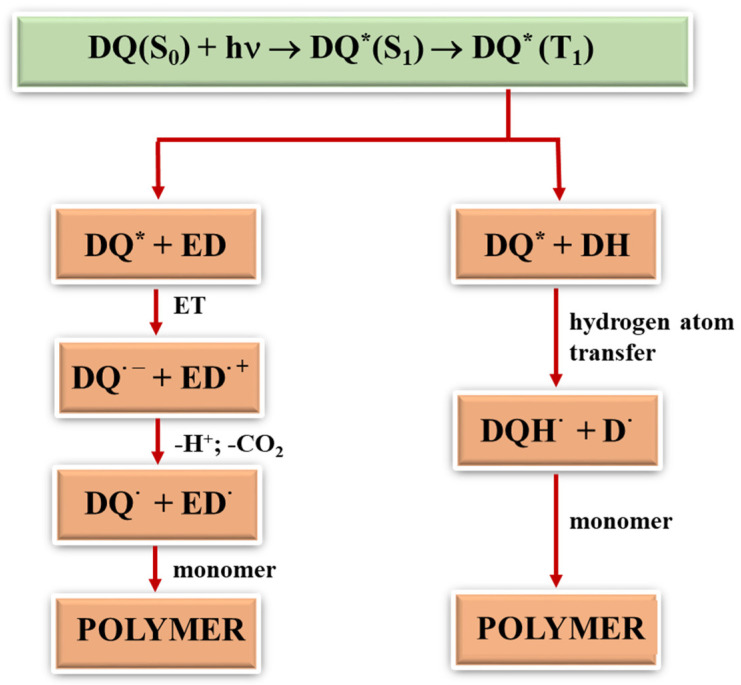
General mechanism of formation of radicals initiating polymerization; (*) the symbol denotes the excited state of the tested dyes (DQ).

Generally, free radicals that initiate photopolymerization are formed by the homolytic cleavage of bonds after the input of significant amounts of energy or by the electron transfer. In the latter, not only does light play a vital role, but so does the type of the second component in the photoinitiating composition since radicals are formed in bimolecular processes. During photoinduced intermolecular electron transfer (PET) processes [24,41,42], the excited electron is transferred from an electron donor (co-initiator) to an electron acceptor (dye). This process is effective if both molecules are at the right distance from each other. Therefore, in two-component photoinitiating systems without electrostatic interactions in the ground state, the diffusion and lifetime of the excited state of the molecule play a key role. After the electron transfer process, subsequent reactions occur, i.e., proton transfer, proton transfer and decarboxylation, as well as other reactions that lead to the formation of a radical that initiates polymerization. In addition to the PET mechanism, other classic photochemical reactions may occur to generate radicals that initiate the polymerization reaction, e.g., hydrogen atom transfer [41,55,56]. The dye molecule detaches a hydrogen atom from the co-initiator (MBX), creating two radicals. The resulting radicals can recombine with each other to give various products (Figure 5). The rate of the hydrogen atom transfer depends on the energy needed to break the bond between the hydrogen atom and the rest of the co-initiator molecule. The most commonly used hydrogen atom donors are amines, alcohols and thiols [57].

As shown in Figure 1 and Figure 2, the tested dyes form a long-lived triplet state, which makes them effective chromophores in two-component photoinitiating systems containing as co-initiator (electron donor or hydrogen donor) PhTAA, PhAA, PhPA, DMA, PhIAA, PhTACA and MBX. Due to the possibility of applying these compounds in dentistry, camphorquinone—a photoinitiator for dentistry—was used for comparative purposes.

#### 2.3.1. Photoinitiation by the Mechanism of Intermolecular Electron Transfer Influence of the Photoinitiator Structure

**Figure 6:**
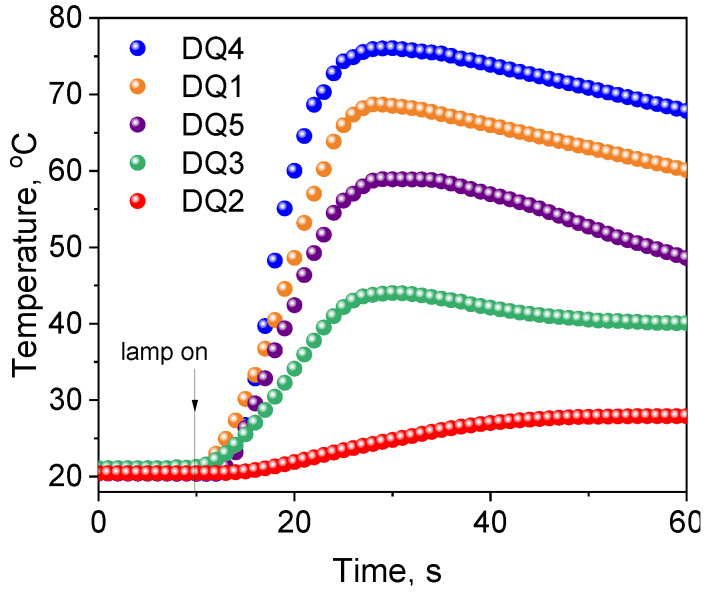
Kinetic curves of TMPTA polymerization photoinitiated by synthesized dyes in pair with electron donor—thiophenoxyacetic acid (0.1 M). The light intensity emitted by the dental lamp was 20 mW cm^−2^.

Figure 6 shows exemplary kinetic curves recorded during photoinitiation of free radical polymerization of TMPTA initiated by synthesized dyes in the presence of the electron donor, PhTAA. The analysis of the kinetic curves and the initial rates of photoinitiated polymerization collected in Table 4 prove that the efficiency of TMPTA photoinitiation depends on the structure of the quinoline and quinoxaline derivatives.

Among the tested dyes, the most effective photoinitiators of TMPTA polymerization were DQ1 and DQ4. When designing the structures of these compounds, the need to generate a long-lived triplet state with high efficiency was taken into account, because via this state, radicals that initiate the photopolymerization reaction are effectively formed. The kinetic equations describing the photopolymerization rate [27] indicate that it depends, among other parameters, on the quantum yield of triplet state formation. Photocurable compositions containing a quinoline derivative (DQ2) have a poor ability to initiate the TMPTA polymerization process. Modification of its structure by introducing an additional nitrogen atom result in an increase in the quantum yield of the triplet state formation by as much as 90 times (DQ2 Φ_T_ = 0.006 vs. DQ4 Φ_T_ = 0.54), and thus an increase in the photopolymerization rate by approximately 20 times. However, indenoquinoxaline DQ5 has almost three times higher quantum yield of triplet state formation compared to indoloquinoxaline DQ3, which initiates the acrylate polymerization reaction less effectively. The introduction of an additional methyl group into the structure of the quinoxaline derivative (DQ3 vs. DQ4) results in an increase in the quantum yield of the triplet state formation by six times. The methyl group is an electron-donating substituent capable of donating non-bonding electrons to the conjugated double bond of the aromatic ring (partial charge transfer occurs from the carbon atom to the benzene ring). This causes the bathochrome effect observed in the long-wavelength band of the absorption spectrum (DQ3—386 nm, DQ4—405 nm). Therefore, DQ4 absorbs visible light more effectively. As a result, this dye has better photoinitiating abilities than DQ3.

##### Influence of Co-Initiator Structure

**Figure 7:**
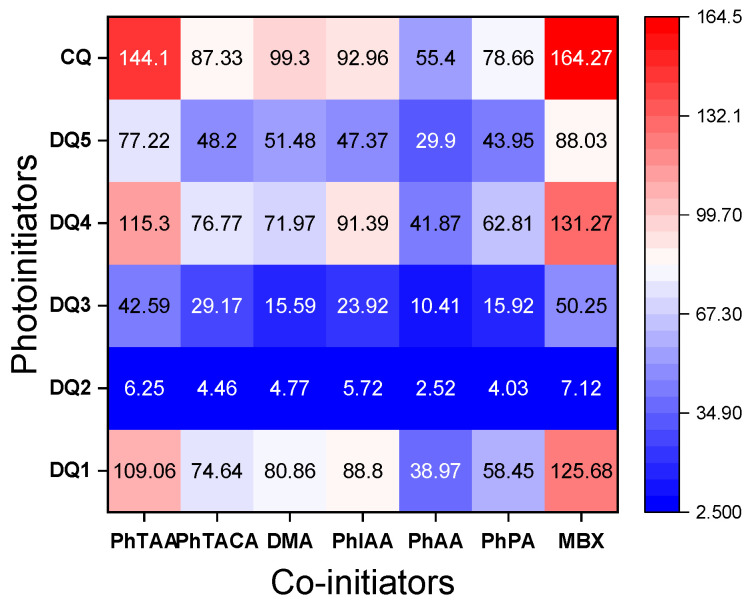
Heat maps of polymerization rates (μmol s^−1^) obtained during photoinitiated polymerization using tested dyes and co-initiators (0.1 M). The light intensity of the dental lamp was 20 mW cm^−2^.

The data presented in Figure 6 and Figure 7 and Table 4 confirm that the structure of the co-initiator has a decisive influence on the initial rate of TMPTA radical polymerization. The selection of electron donors was based on our many years of research on the photoinitiated polymerization process [19,23,29,57,58]. A thorough analysis of the presented results confirms that the presence of an oxygen atom in the electron donor structure (PhAA and PhPA) reduces the initial photopolymerization rate compared to co-initiators containing a sulfur atom (PhTAA and PhTACA) and a nitrogen atom (PhIAA and DMA). This is associated with a more efficient PET process and the different reactivity of radicals generated in secondary reactions following the electron transfer process. The following polymerization initiating radicals can be formed from the co-initiators: from phenoxyacetic acid—$C_{6}H_{5}O\dot{C}HCOOH$ and $C_{6}H_{5}O\dot{C}H_{2}$, from thiophenoxyacetic acid—$C_{6}H_{5}S\dot{C}HCOOH$ and $C_{6}H_{5}S\dot{C}H_{2}$ and from *N*-phenyiminodiacetic acid -$C_{6}H_{5}NH\dot{C}{\left(COOH\right)}_{2}$ and $C_{6}H_{5}NH\dot{C}H_{2}$ [58,59,60,61,62]. Analysis of the products obtained from *N*,*N*-dimethylaniline under photoreductive conditions indicates the presence of *N*-methylformanilide, *N*-methylaniline and trace amounts of other compounds [63], while thioanisole, thiophenol, CO_2_ and diphenyl-disulfide can be formed from thiophenoxyacetic acid [30,64]. Moreover, the decomposition of phenoxyacetic acid produces anisole, phenol, CO_2_ and 1,2-diphenoxyethane [65,66], while *N*-methyl-aniline, aniline, formanilide, CO_2_, as well as dianilinomethane and *N*-methyleneaniline are obtained from *N*-phenyliminodiacetic acid [64,65,66,67].

The second carboxyl group present at the heteroatom is also responsible for the increase in the polymerization rate of TMPTA in systems containing PhIAA as an electron donor. This is confirmed by the fact that in the initiation mechanism based on carboxylic acids, after the electron transfer process, subsequent reactions involve proton transfer and decarboxylation, which lead to the formation of a radical initiating polymerization (Figure 5) [60]. Thus, the presence of two carboxyl groups in the electron donor molecule may lead to the formation of a diradical.

Furthermore, the analysis of the data collected in Table 4 and heat maps (Figure 7) shows that the synthesized dyes are effective photoinitiators of the polymerization reaction, also initiated by the hydrogen atom transfer mechanism. The photopolymerization rates obtained for light-cured compositions containing 2-mercaptobenzoxazole (MBX) as a hydrogen atom donor are comparable to those containing the electron donor PhTAA.

To sum up, the obtained quinoline and quinoxaline derivatives can be used as visible light photoinitiators in systems containing both electron donors and hydrogen atom donors as co-initiators. In the tested systems, radicals initiating TMPTA polymerization are formed as a result of intermolecular electron transfer, followed by the proton transfer between the components of the radical ion pair or decarboxylation depending on the solvent polarity. The second mechanism involves the hydrogen atom transfer from 2-mercaptobenzoxazole to the excited dye.

#### 2.3.2. Control Tests Using Commercial Compounds

**Figure 8:**
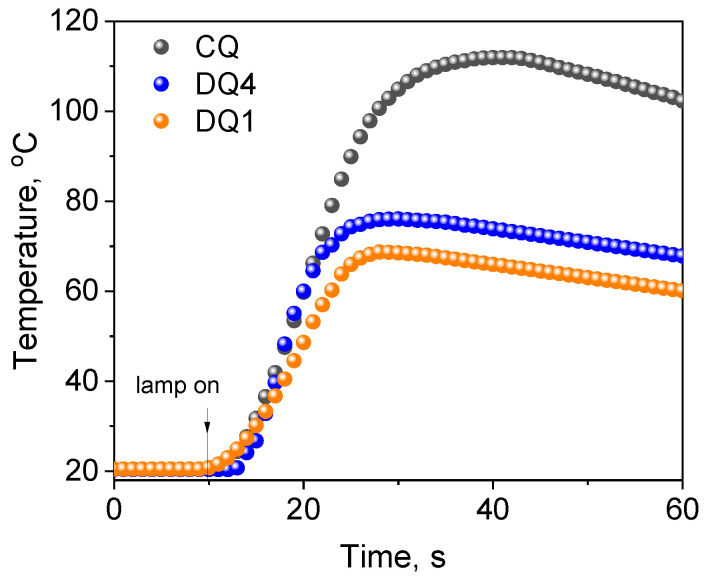
Comparison of the photoinitiating abilities of systems containing quinoline[2,3-b]-1*H*-imidazo[1,2-a]pyridinium bromide (DQ1) and 6-methyl-6*H*-indolo[2,3-b]quinoxaline (DQ4) as photoinitiator to camphorquinone (CQ). The tested photoinitiating systems have the same co-initiator (phenylthio)acetic acid (PhTAA). Kinetic curves were recorded during measurements of temperature changes during TMPTA polymerization photoinitiated by DQ1–PhTAA, DQ4-PhTAA and CQ–PhTAA pairs. The co-initiator concentration was 0.1 M and the light intensity of the dental lamp was 20 mW/cm^2^.

To verify the photoinitiating abilities of the tested systems (quinoline and quinoxaline derivatives (DQ1–DQ5)—co-initiators), the initial rates of free radical polymerization were determined and compared with the rate obtained for the system based on the commercial CQ photoinitiator used in dentistry (Figure 8). To conduct the experiment precisely, the same conditions were maintained, i.e., the number of absorbed photons. Therefore, the concentration of CQ (approx. 0.675 M) in the composition was approximately 500 times higher than that of the tested sensitizers due to the lower molar absorption coefficient in the visible region (8 M^−1^ cm^−1^ vs. approximately 4000 M^−1^ cm^−1^ at 400 nm). As a result, thick polymer layers (3 mm) were obtained for the new systems, which do not contain significant amounts of unreacted photoinitiator and products formed during its decomposition.

Moreover, the temperature increases during polymerization photoinitiated by quinoline/quinoxaline derivative—thiophenoxyacetic acid systems was lower than for the commercial CQ photoinitiator, which is particularly important from the viewpoint of the potential application of these systems in dentistry. The data presented in Table 4 and Figure 8 clearly indicate that the initial TMPTA polymerization rates for systems containing DQ1 and DQ4 are similar to the data obtained for CQ. Hard polymer enamel is obtained after approximately 25 s of exposure (Figure 8).

#### 2.3.3. Kinetic and Thermodynamic Conditions of the Polymerization Process

**Figure 9:**
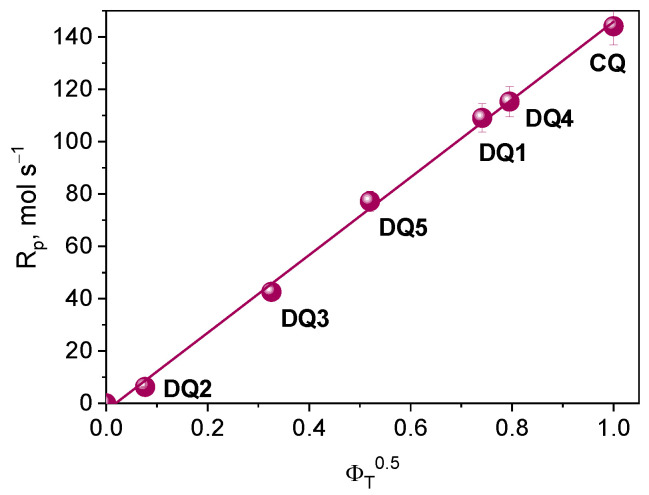
Dependence of the initial rate of photoinitiated TMPTA polymerization on the square root of the quantum yield of triplet state formation.

According to the kinetic equations describing the photopolymerization rate, the formation of radicals occurs in the triplet state of the photoinitiator. Considering this assumption, for the tested photoinitiating systems, the initial rate of photoinitiated TMPTA (*R_p_*) polymerization should depend linearly on the square root of the quantum yield of triplet state formation. Based on the correlation shown in Figure 9, it can be concluded that the electron transfer process from the electron donor (PhTAA) to the photoinitiator (DQ1–DQ5) takes place in the excited triplet state.

**Figure 10:**
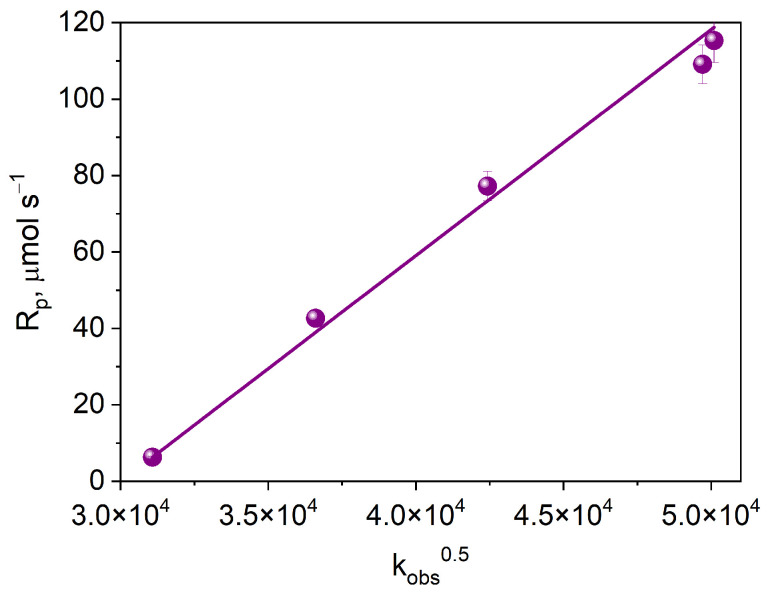
Dependence of the initial rate of photoinitiated TMPTA polymerization on the square root of the electron transfer rate constant.

Figure 10 shows the dependence of the initial rate of photoinitiated TMPTA polymerization on the square root of the electron transfer rate constant. The observed linear correlation indicates that the process limiting the initial rate of free radical polymerization is the intermolecular electron transfer process.

Radical polymerization proceeding through the mechanism of intermolecular electron transfer is a multi-stage process, the main of which is the electron transfer from an electron donor molecule to an electron acceptor molecule in the excited state. The photoinduced electron transfer process is limited by thermodynamic factors, including the free energy of activation for the electron transfer process (PET), Δ*G_el_*. The Δ*G_el_* value can be determined experimentally using the Rehm–Weller equation [68,69]:

(3) $$∆G_{el}=E_{ox}-E_{red}-\frac{Ze^{2}}{\varepsilon a}-E_{T}^{00}$$

where: *E_ox_* is the oxidation potential of the co-initiator, *E_red_* is the reduction potential of the dye, $E_{T}^{00}$ is the energy of the excited state (transition energy T_1_ → S_0_)

To calculate the Δ*G_el_* value, the oxidation potential of the co-initiator—PhTAA (*E_ox_* = 1.450 V), the reduction potential of electron acceptors (Table 1) and the transition energy T_1_ → S_0_ should be determined. The data are summarized in Table 1. The negative Δ*G_el_* values indicate that the photoinduced electron transfer process is thermodynamically allowed.

**Figure 11:**
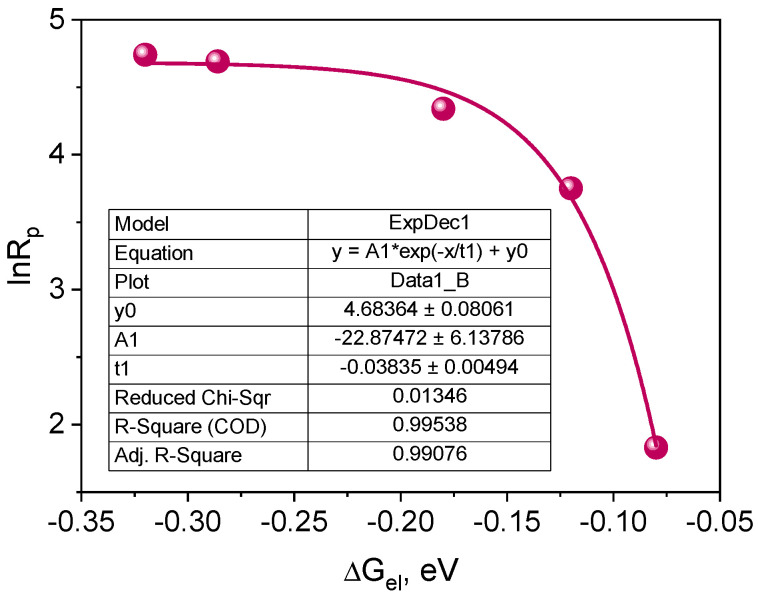
Dependence of the initial polymerization rate on the free energy of activation for the electron transfer process (PET) from the electron donor to the excited triplet state of the synthesized dyes.

According to Equation (4), if the intermolecular electron transfer process between the excited dye molecule and the electron donor molecule is a process limiting the polymerization rate, a parabolic relationship between the polymerization rate (*R_p_*) and the free energy of activation (Δ*G_el_*) for the PET process should be observed [27,70,71,72].

(4) $$lnR_{p}=A-\frac{(\lambda +\Delta G_{el}{)}^{2}}{8\lambda RT}$$

where: *A* for the initial polymerization time is:

(5) $$A=lnk_{p}-0.5\;lnk_{t}+1.5\ln\left[M\right]+0.5lnI_{A}$$

where: *k_p_* and *k_t_* are the polymerization rate and chain termination constant, respectively, [*M*] is the monomer concentration, *I_A_* is the intensity of the absorbed light, *λ* is the energy necessary to achieve the transition states of both the excited molecule and the solvent molecule. The correlation resulting from Equation (4) is shown in Figure 11.

The plot presented in Figure 11 indicates that the correlation between the initial polymerization rate and the free energy of activation for the electron transfer process (PET) is a fragment of a parabola. Therefore, it can be concluded that the tested photoredox pairs behave in accordance with the classical theory of the photoinduced electron transfer process [70,72]. The PET process is a rate-limiting polymerization process. Experimental points are located in the so-called normal Marcus area, i.e., the area in which the rate of the process increases as its driving force increases [27,70,72,73].

## 3. Materials and Methods

### 3.1. Reagents

Reagents for dye synthesis were purchased from Alfa Aesar Co., Ward Hill, MA, USA (ninhydrin, 1,2-diaminobenzene) and Merck, Rahway, NJ, USA (ethyl bromoacetate, 2-indolinone, isatin, 1-methylisatin, 2-aminopyridine, 2-aminobenzaldehyde). Solvents used either for synthesis or spectroscopic measurements, i.e., acetic acid, isopropanol, ethanol, methanol, ethyl acetate, 1-methyl-2-pyrrolidinone (MP), chloroform, deuterated chloroform (CDCl_3_) and dimethyl sulfoxide (DMSO-*d*_6_) were provided by Merck. Monomer, trimethylolpropane triacrylate (TMPTA), commercial photoinitiator, camphorquinone (CQ) and electron donors: (phenylthio)acetic acid (PhTAA), phenylacetic acid (PhAA), 2-phenylpropionic acid (PhPA), *N*,*N*-dimethylaniline (DMA), 3-(phenylthio)acrylic acid (PhTACA) and hydrogen donor 2-mercaptobenzoxazole (MBX) were obtained from Sigma-Aldrich Co., St. Louis, MO, USA while *N*-phenyliminodiacetic acid (PhIAA) was from Lancaster, Kenilworth, NJ, USA. Thin layer chromatography plates (DC-Plastikfolien Silica gel 60 F254, 0.2 mm) from Merck Co. Rahway, NJ, USA.

**Figure 12:**
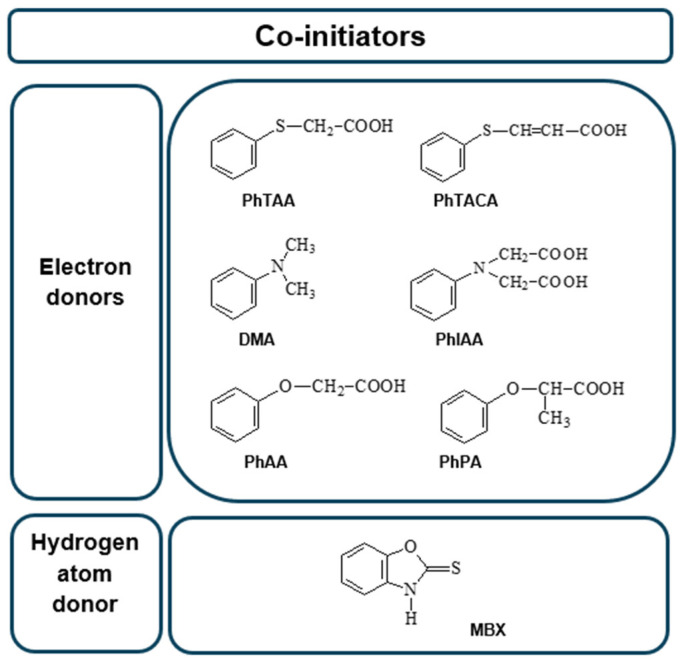
Structures of co-initiators used in the photoinitiating composition.

The structures of the co-initiators used are shown in Figure 12. They are divided into two groups, i.e., electron donors and hydrogen atom donors.

### 3.2. Synthesis

**Figure 13:**
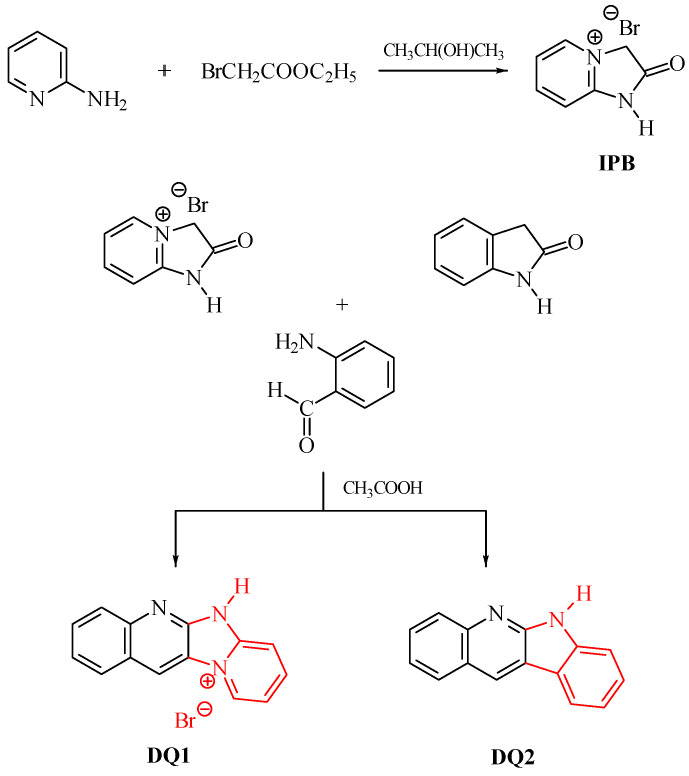
Synthesis of quinoline derivatives DQ1–DQ2; red color indicates the main modification of the dye structure.

**Figure 14:**
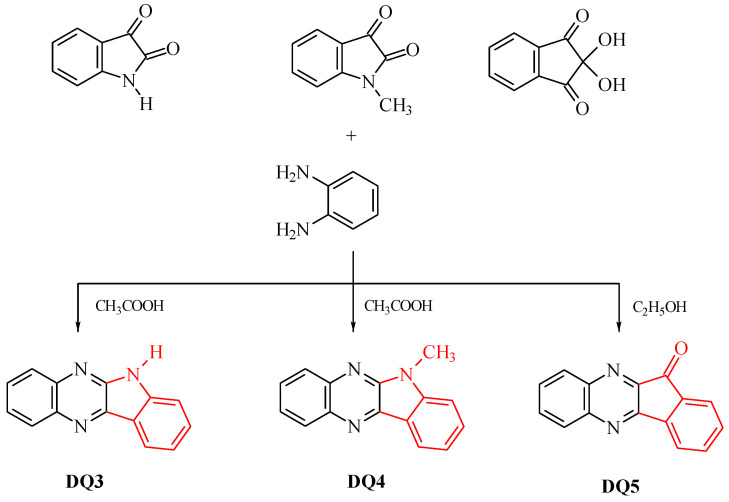
Synthesis of quinoxaline derivatives DQ3–DQ5; red color indicates the main modification of the dye structure.

The dyes were synthesized by the reactions shown in Figure 13 and Figure 14. The methods for synthesizing the tested compounds described in this work, although based on literature data, have been modified and improved for simplicity. The available literature describes their syntheses based on other reagents or other solvents with different yields [74,75]. In some cases, other techniques were also used, e.g., microwave [76,77].

For example, the synthesis of the DQ1 dye reported by us has not been described in the literature. In Van Dormael’s work [78], only the first stage of the synthesis is described, leading to the intermediate product 2-oxo-2,3-dihydro-1*H*-imidazo[1,2-a]pyridinium bromide. Moreover, the author proposes carrying out the reaction without adding a solvent. Due to the high exothermicity of this process, it is very troublesome. Therefore, we tested a large group of solvents and found that the reaction was most effective with the addition of 2-propanol. Moreover, unlike the work we published in *Polymer* in 2007 [79], the second stage of the synthesis was completely refined and shortened. In a previous work [79], 2-oxo-2,3-dihydro-1H-imidazo[1,2-a]pyridinium bromide was used as an intermediate, which was condensed with 2-nitrobenzaldehyde and then reduced with iron. Unfortunately, the final product was obtained with unsatisfactory yield. In this work, 2-aminobenzaldehyde was used as a reagent, which in the presence of glacial acetic acid very easily condenses with 2-oxo-2,3-dihydro-1*H*-imidazo[1,2-a]pyridinium bromide to give the desired compounds. The developed method of synthesizing the tested compounds (DQ1–DQ5) using glacial acetic acid as a solvent is very simple. The reaction is carried out under reflux at normal pressure without the use of microwaves and the products are obtained in good yields.

The progress of the reactions was monitored by thin-layer chromatography on silica gel 60F 254 using chloroform:methanol (9:1 *v*/*v*) (DQ1, DQ2, DQ3, DQ4) and chloroform (DQ5) as an eluent. The products were identified spectroscopically. The ^1^H and ^13^C NMR spectra are shown as images before Figure S1 in Supplementary Materials.

DQ1: Quinoline[2,3-b]-1*H*-imidazo[1,2-a]pyridinium bromide

The dye quinoline[2,3-b]-1*H*-imidazo[1,2-a]pyridinium bromide (DQ1) was obtained in a two-step reaction according to the following procedure.

Step 1: 2-oxo-2,3-dihydro-1*H*-imidazo[1,2-a]pyridinium bromide (IPB)

In the first stage, to obtain an intermediate product—2-oxo-2,3-dihydro-1*H*-imidazo[1,2-a]pyridinium bromide (IPB), 4.7 g (0.05 mol) 2-aminopyridine and 25 mL of 2-propanol. At a boiling point of approximately 82 °C, 6.2 g (0.05 mol) of ethyl chloroacetate were added dropwise. After complete addition of ethyl bromoacetate, the reaction mixture was heated for approximately 30 min. The separated precipitate of 2-oxo-2,3-dihydro-1*H*-imidazo[1,2-a]pyridinium bromide was filtered and then washed twice with 5 mL of acetone. The obtained product was crystallized from 40 mL of methanol to obtain 3.5 g (45%) of crystals, C_7_H_7_N_2_OBr, 215.04 g/mol, m.p. 230–232 °C [78,79].

^1^H NMR (400 MHz, DMSO-*d*_6_) δ (ppm): 8.80–8.77 (d, ^3^*J_H,H_* = 8.0 Hz, 1H), 8.44–8.35 (t, 1H), 7.60–7.59 (d, ^3^*J_H,H_* = 8.0 Hz, 1H), 7.57–7.52 (t, 1H), 5.29 (s, 2H, CH_2_).

^13^C NMR (200 MHz, DMSO-*d*_6_) δ (ppm): 169.6, 153.3, 147.0, 139.4, 118.8, 110.7, 55.3.

Step 2

In the second stage, 2.15 g (0.01 mol) of 2-oxo-2,3-dihydro-1*H*-imidazo[1,2-a]pyridinium bromide and 50 mL of glacial acetic acid were introduced into a round-bottomed flask equipped with a reflux condenser and a stirrer. The mixture was stirred and 1.21 g (0.01 mol) of 2-aminobenzaldehyde was added at room temperature. The reaction mixture was heated at reflux for 3 h to give 2.04 g (68%) of crystals. The obtained product was crystallized from 50 mL of methanol, C_13_H_10_N_3_Br, 300.15 g/mol, m.p. 264–265 °C [78,79].

^1^H NMR (400 MHz, DMSO-*d*_6_) δ (ppm): 7.11–7.15 (d, ^3^*J_H,H_* = 8.0 Hz, 1H), 7.28–7.35 (t, 1H), 7.38–7.46 (t, 1H), 7.89–7.93 (d, ^3^*J_H,H_* = 8.0 Hz, 1H), 8.00–8.03 (d, ^3^*J_H,H_* = 8.0 Hz, 1H), 8.14–8.18 (t, 1H), 8.25–8.29 (d, ^3^*J_H,H_* = 8.0 Hz, 1H), 8.53 (s, 1H), 9.40–9.58 (t, 1H), 12.55(NH, 1H).

^13^C NMR (200 MHz, DMSO-*d*_6_) δ (ppm): 152.1, 148.9, 140.6, 132.8, 130.8, 129.7, 126.1, 125.8, 124.3, 122.4, 122.3, 115.4, 115.1.

DQ2; 6*H*-indolo[2,3-b]quinoline

6*H*-indolo[2,3-b]quinoline (DQ2) was obtained by refluxing 1.33 g (0.01 mol) 2-indolinone with 1.21 g (0.01 mol) 2-aminobenzaldehyde in the presence of glacial acetic acid (40 mL). The isolated product was crystallized from a 1:1 *v*/*v* mixture of dimethylformamide and ethanol to obtain 1.72 g (79%) of light yellow crystals, C_15_H_10_N_2_, 218.25 g/mol, m.p. 342–345 °C [80,81].

^1^H NMR (400 MHz, DMSO-*d*_6_) δ (ppm): 11.68 (s, 1H), 9.04 (s, 1H), 8.27–8.23 (d, ^3^*J_H,H_* = 8.0 Hz, 1H), 8.12–8.07 (d, ^3^*J_H,H_* = 8.0 Hz, 1H), 7.98–7.94(d, ^3^*J_H,H_* = 8.0 Hz, 1H), 7.50–7.55 (t, 1H), 7.52–7.42 (m, 3H), 7.29–7.21 (t, 1H).

^13^C NMR (200 MHz, DMSO-*d*_6_) δ (ppm): 146.3, 144.4, 140.6, 140.2, 139.0, 131.7, 129.5, 129.2, 127.95, 126.4, 122.7, 121.1, 119.4, 112.4.

DQ3; 6*H*-indolo[2,3-b]quinoxaline

6*H*-indolo[2,3-b]quinoxaline (DQ3) was obtained by refluxing (approx. 1 h) 1.47 g (0.01 mol) isatin with 1.08 g (0.01 mol) 1,2-diaminobenzene in glacial acetic acid (60 mL). The isolated product was crystallized from a mixture of dimethylformamide and ethanol (1:1 *v*/*v*) to obtain 1.65 g (75%) of light yellow crystals, C_14_H_9_N_3_, 219.24 g/mol, m.p. 297–298 °C [74,82,83,84].

^1^H NMR (400 MHz, DMSO-*d*_6_) δ (ppm): 12.05 (s, 1H), 8.35–8.37 (d, ^3^*J_H,H_* = 8.0 Hz, 1H), 8.25–8.27 (m, 1H), 8.07–8.09 (m, 1H), 7.79–7.83 (m, 1H), 7.70–7.75 (m, 2H), 7.58–7.60 (d, ^3^*J_H,H_* = 8.0 Hz, 1H), 7.36–7.40 (m, 1H).

^13^C NMR (200 MHz, DMSO-*d*_6_) δ (ppm): 146.3, 144.4, 140.6, 140.2, 139.0, 131.7, 129.5, 129.2, 127.9, 126.4, 122.7, 121.1, 119.4, 112.4.

DQ4; 6-methyl-6*H*-indolo[2,3-b]quinoxaline

6-methyl-6*H*-indolo[2,3-b]quinoxaline (DQ4) was prepared by refluxing 1.61 g (0.01 mol) 1-methylisatin with 1.08 g (0.01 mol) 1,2-diaminobenzene in glacial acid vinegar (60 mL). The isolated product was crystallized from a mixture of dimethylformamide and ethanol (1:1 *v*/*v*) to obtain 1.88 g (81%) of light yellow crystals, C_15_H_11_N_3_, 233.26 g/mol, m.p. 146–147 °C [82,85,86].

^1^H NMR (400 MHz, CDCl_3_) δ (ppm): 8.52–8.48 (d, ^3^*J_H,H_* = 8.0 Hz, 1H), 8.34–8.30 (d, ^3^*J_H,H_* = 8.0 Hz, 1H), 8.18–8.13 (d, ^3^*J_H,H_* = 8.0 Hz, 1H), 7.78–7.69 (m, 3H), 7.51–7.41 (m, 1H), 7.37–7.26 (m, 1H), 4.03 (CH_3_, 3H).

^13^C NMR (200 MHz, CDCl_3_) δ (ppm): 145.9, 145.4, 140.7, 140.3, 132.1, 131.0, 129.3, 127.7, 127.5, 126.8, 122.1, 121.5, 110.3, 109.4, 27.8.

DQ5; 11*H*-indeno[1,2-b]qunioxalin-11-on

11*H*-indeno[1,2-b]qunioxalin-11-one (DQ5) was prepared by refluxing 1.78 g (0.01 mol) ninhydrin with 1.08 g (0.01 mol) 1,2-diaminobenzene in ethanol (50 mL). Three drops of acetic acid were added to the reaction mixture and heated at reflux for 3 h. The isolated product was crystallized from dimethylformamide to obtain 2.10 g (91%) of light yellow crystals, C_15_H_8_N_2_O, 232.24 g/mol, m.p. 224–225 °C [82,85,86].

^1^H NMR (400 MHz, DMSO-*d*_6_) δ (ppm): 8.20–8.14 (t, 2H), 8.11–8.09 (d, *^3^J_H,H_* = 8 Hz, 1H), 7.95–7.84 (m, 4H), 7.74–7.70 (t, 1H).

^13^C NMR (200 MHz, DMSO-*d*_6_) δ (ppm): 189.8, 156.9, 150.3, 142.6, 142.3, 141.5, 137.4, 137.1, 133.2, 132.9, 131.4, 130.8, 129.8, 124.7, 122.7.

### 3.3. Methods

Electronic absorption spectra were recorded on a Shimadzu UV-Vis Multispec-1501 spectrophotometer, Kioto, Japan, in ethyl acetate solution. Electronic emission spectra were recorded on a Hitachi F-4500 spectrophotometer, Tokio, Japan. Fluorescence spectra were recorded at room temperature in 2-methyltetrahydrofuran. The fluorescence quantum yield was determined by a comparative method using a solution of 9-methylanthracene [41] in 2-methyltetrahydrofuran as a standard. Phosphorescence spectra were recorded in 2-methyltetrahydrofuran at liquid nitrogen temperature.

The reduction potentials of the synthesized dyes were determined in a 0.1 M solution of tetrabutylammonium perchlorate in anhydrous acetonitrile, based on cyclic voltammograms recorded on an MTM Model EA9C-4z cyclovoltammeter, Electroanalytical MTM System (Krakow) Model EA9C-4z, EA, Krakow, Poland. A platinum disk electrode was used as the working electrode and a platinum wire and an Ag/AgCl electrode were used as the auxiliary and reference electrodes, respectively.

The kinetics of photoinitiated polymerization was studied using the microcalorimetric method [30,35,87]. The polymerizing compositions contained 0.9 g of TMPTA as monomer, 0.1 mL of 1-methyl-2-pyrrolidone (MP), from 1.35 × 10^−3^ to 1.8 × 10^−3^ M (depending on the molar absorption coefficient) of synthesized dyes as photoinitiators (electron acceptors) and 0.1 M of an electron donor as a co-initiator. The reference formulation did not contain an electron donor. The photocurable composition was placed in a Teflon ring with a diameter of 10 mm and a thickness of 3 mm, protected on one side with a glass plate. A thermocouple (stainless steel, type K, Φ4 mm, RTD Thermometer Delta OHM HD 2107.1, Delta OHM, Padova, Italy) was placed in it from the top so that its tip was in contact with the surface of the glass plate. The sample was irradiated through the bottom glass plate with blue light from a dental LED lamp (Cromalux 75 Mega Phisik Dental, 390–500 nm, MEGA-PHYSIK GMBH & CO, Rastatt, Germany). Its intensity was checked with a Coherent Field Master meter and amounted to 20 mW/cm^2^. The distance of the sample from the light source was the same for all measurements. The time needed to stabilize the temperature in the sample before irradiation was 10 s and was the same for all experiments. Temperature changes during the polymerization process were recorded every 1 s with an accuracy of ±0.1 °C using a recorder (Delta OHM HD 40.1, Padova, Italy) connected to a thermocouple. Three measurements were performed for each of the tested materials. A commercial photoinitiator—camphorquinone (CQ) was used to assess the effectiveness of initiating the polymerization reaction by the tested systems. Its concentration was 0.675 M.

The lifetime of the excited triplet state of the synthesized dyes was determined in acetonitrile based on transient absorption spectra recorded on nanosecond laser flash photolysis using an LSK 60 Laser Flash Photolytics camera (Applied Photophysics, Leatherhead, UK). A pulsed laser emitting radiation with a wavelength of 355 nm from Lambda Phisik/model LPY 150, Saxonburg, PA, USA) was used to excite the sample. The energy of the laser pulses was 65 mJ.

## 4. Conclusions

The paper focused on two-component photoinitiating systems based on quinoline and quinoxaline dyes combined with acetic acid derivatives for application in dental filling materials. To obtain effective photoinitiators operating in the visible region, the parent structure of quinoline (DQ2) was modified to the quinoxaline derivatives. Therefore, the synthesized compounds differ in the number of nitrogen atoms (DQ1–DQ5), the presence of a methyl substituent (DQ4), or an oxygen atom (DQ5). Their full spectral characteristics based on NMR, UV-Vis, fluorescence and phosphorescence, along with a description of their synthesis, are presented. The electronic absorption spectra of these dyes are characterized by an intense absorption band located in the range of 320–350 nm and a second, less intense absorption band shifted by ca. 35–50 nm to the red. The results of steady-state and time-resolved spectroscopic studies confirm the formation of the triplet state by the tested dyes. Based on the recorded transient absorption spectra, the lifetime and quantum yield of the triplet state were determined. The revealed physicochemical properties of the tested dyes allowed their use as initiators in compositions photoinitiated with light emitted by a dental lamp.

These compounds turned out to be effective photoinitiators of free radical polymerization of TMPTA in the visible light range, induced by dental lamp light in the presence of an electron donor or a hydrogen atom. Good photoinitiation abilities have been confirmed by comparative tests with systems containing a commercial photoinitiator used in dentistry—camphorquinone (CQ). Two of the obtained compounds, a quinoline derivative (DQ1) and a quinoxaline derivative (DQ4), used in light-cured compositions, indicate similar values of the initial photopolymerization rate of acrylates to the data obtained for CQ. The high rate of the chain reaction and short polymerization time confirm that the new two-component initiating systems can be used in much lower concentrations, without slowing down the speed of obtaining polymer materials.

## Figures and Tables

**Table 1:** Spectroscopic and electrochemical properties, quantum yield of triplet state formation and triplet state lifetime of the tested dyes.

Dyes	**^a^** $\lambda_{\max}^{Abs.}$ (nm)	*ε* (M^−1^ cm^−1^)	**^b^** $\lambda_{\max}^{Fl}$ (nm)	Φ*_Fl_*	$\lambda_{max}^{Ph}$ (nm)	$E_{T}^{00}$ (eV)	*E_red_* (V)	^c^ Δ*G_el_* (eV)
DQ1	387	2610	447	0.230	526	2.79	−1.054	−0.286
	408	3040	477		542			
	432	2360	511		572			
DQ2	316	14,400	406	0.176	510	2.51	−0.98	−0.08
	330	20,150						
	367	3980						
DQ3	334	14,100	465	0.039	543	2.74	−1.17	−0.12
	350	16,300						
	386	4200			588			
DQ4	334	14,100	476	0.040	515	2.95	−1.18	−0.32
	351	16,500						
	405	3400			561			
DQ5	287	34,600	443	0.123	529	2.72	−1.09	−0.18
	381	3500						
CQ	472	40	-	-	-	-	-	-

^a^ measured in ethyl acetate, ^b^ measured in 2-methyltetrahydrofuran, ^c^ Δ*G_el_* calculated for PhTAA (*E*_ox_ = 1.450 V) as co-initiator.

**Table 2:** Quantum yield of triplet state formation (Φ_T_) for the tested dyes and triplet state lifetime (τ_T_).

Dyes	Φ_T_	τ_T_, μs
DQ1	0.5490	1.52
DQ2	0.0060	0.74
DQ3	0.1061	1.00
DQ4	0.6320	1.58
DQ5	0.2704	1.31

**Table 3:** Quenching constants of the triplet state of the tested dyes (*k_q_*).

Co-Initiators	*k_q_* × 10^−8^, M^−1^ s^−1^				
	DQ1	DQ2	DQ3	DQ4	DQ5
PhTAA	25.10	9.66	13.40	24.71	18.00
PhTACA	5.20	3.01	3.12	6.25	5.30
DMA	6.94	3.16	3.98	7.09	5.92
PhIAA	16.20	6.62	8.92	17.80	12.30
PhAA	2.79	2.33	2.63	2.65	4.61
PhPA	3.02	2.75	2.98	3.12	5.05
MBX	3.36	1.97	2.01	2.98	3.11

**Table 4:** Initial rate of photoinitiated free radical polymerization of TMPTA initiated by photoredox pairs (R_p_).

Co-Initiators	R_p_, μmol·s^−1^					
	DQ1	DQ2	DQ3	DQ4	DQ5	CQ
PhTAA	109.06	6.25	42.59	115.30	77.22	144.10
PhTACA	74.64	4.46	29.17	76.77	48.20	87.33
DMA	80.86	4.77	15.59	71.97	51.48	99.30
PhIAA	88.80	5.72	23.92	91.39	47.37	92.96
PhAA	38.97	2.52	10.41	41.87	29.90	55.40
PhPA	58.45	4.03	15.92	62.81	43.95	78.66
MBX	125.68	7.12	50.25	131.27	88.03	164.27

## Data Availability

The data are not publicly available apart from the data contained in the article or Supplementary Materials.

## Supplementary Materials

The following supporting information can be downloaded at: https://www.mdpi.com/article/10.3390/ijms25084289/s1.
