# Supplementary material for: Design of Dyes Based on the Quinoline or Quinoxaline Skeleton towards Visible Light Photoinitiators
Source: Int J Mol Sci. 2024 Apr 12;25(8):4289. doi: 10.3390/ijms25084289 (PMC11050332; doi:10.3390/ijms25084289)
Supplement: Supplementary file 1 [file ijms-25-04289-s001.zip › ijms-2949583-supplementary.pdf]

Supporting information

Design of dyes based on the quinoline or quinoxaline skeleton towards visible light photoinitiators

Ilona Pyszka [1], Beata Jędrzejewska [1]

[1] Faculty of Chemical Technology and Engineering, Bydgoszcz University of Science and Technology, 85-326 Bydgoszcz, Poland
* Correspondence: Ilona.Pyszka@pbs.edu.pl; Tel.: +48-52-374-9039

Table of contents **Page**

^{1}H and ^{13}C NMR spectra…………………………………………………………………...	S2
Normalized electronic absorption spectra of synthesized dyes in ethyl acetate – Figure S1…………………………………………………………………………..	S12
Normalized (a) fluorescence and (b) phosphorescence spectra of the synthesized dyes in 2-methyltetrahydrofuran at room temperature – Figure S2……………………………………..…	S13
Electronic absorption spectrum, fluorescence and phosphorescence spectra of synthesized dyes in 2-methyltetrahydrofuran. – Figure S3…………………………………………………	S13
Absorption spectrum of the triplet state of the tested dyes recorded in deoxygenated acetonitrile – Figure S4………………………………………………………………………………..	S14
Kinetic curves of the disappearance of the triplet state recorded for the tested dyes at DQ2 - 430 nm, DQ3 - 370 nm, DQ4 - 360 nm, DQ5 - 340 nm – Figure S5……………………………….	S14

^{1}H spectrum of **IPB**

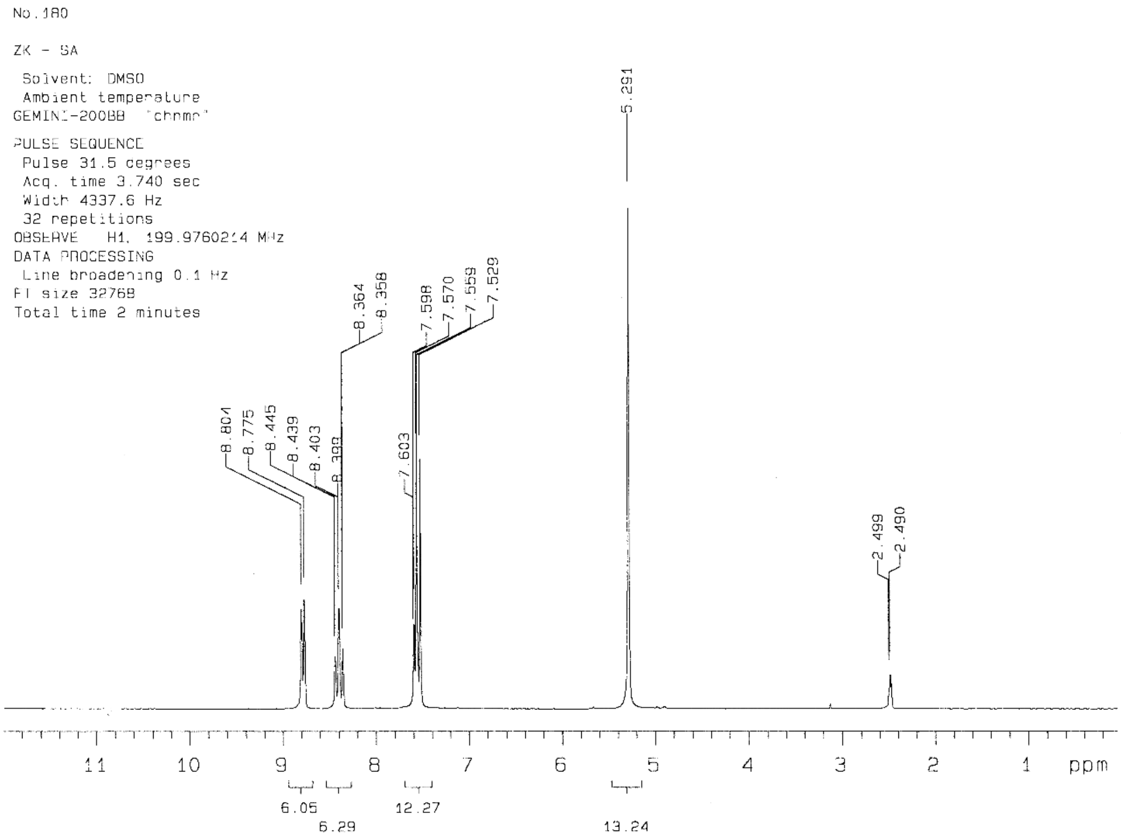

^{13}C spectrum of **IPB**

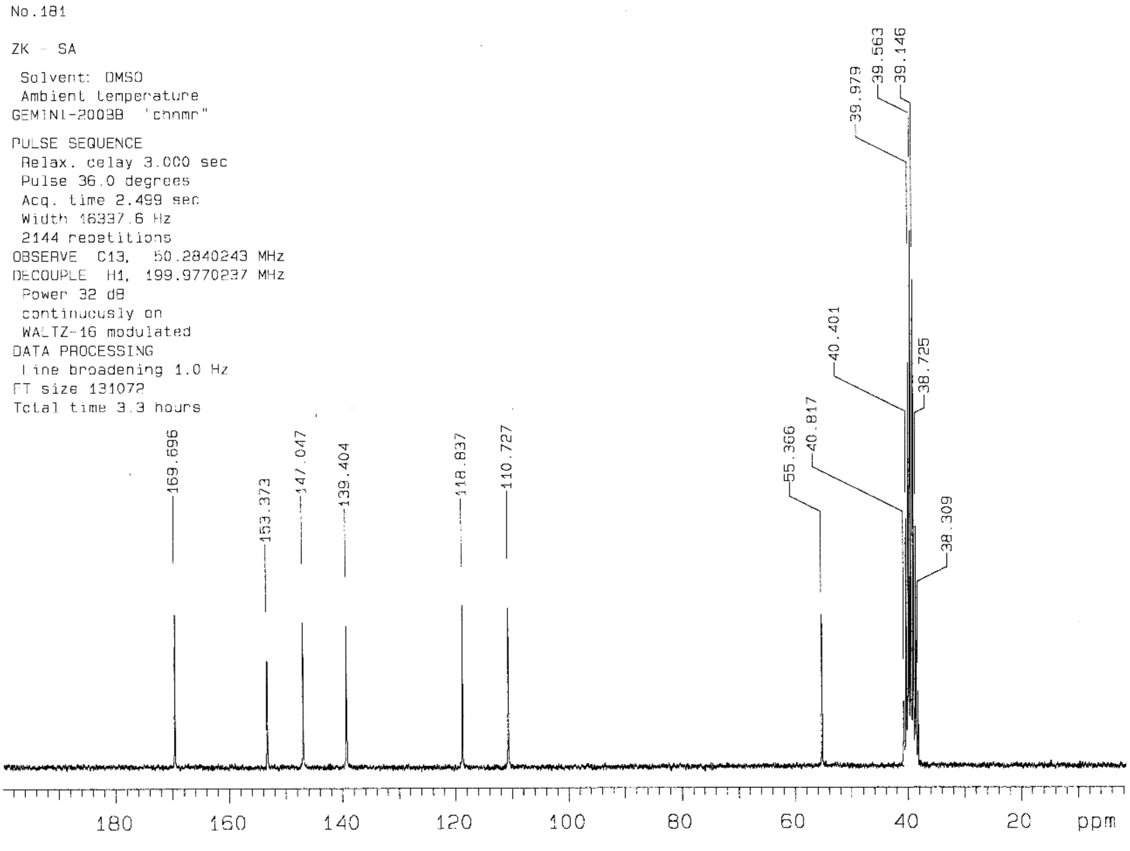

^{1}H spectrum of **DQ1**

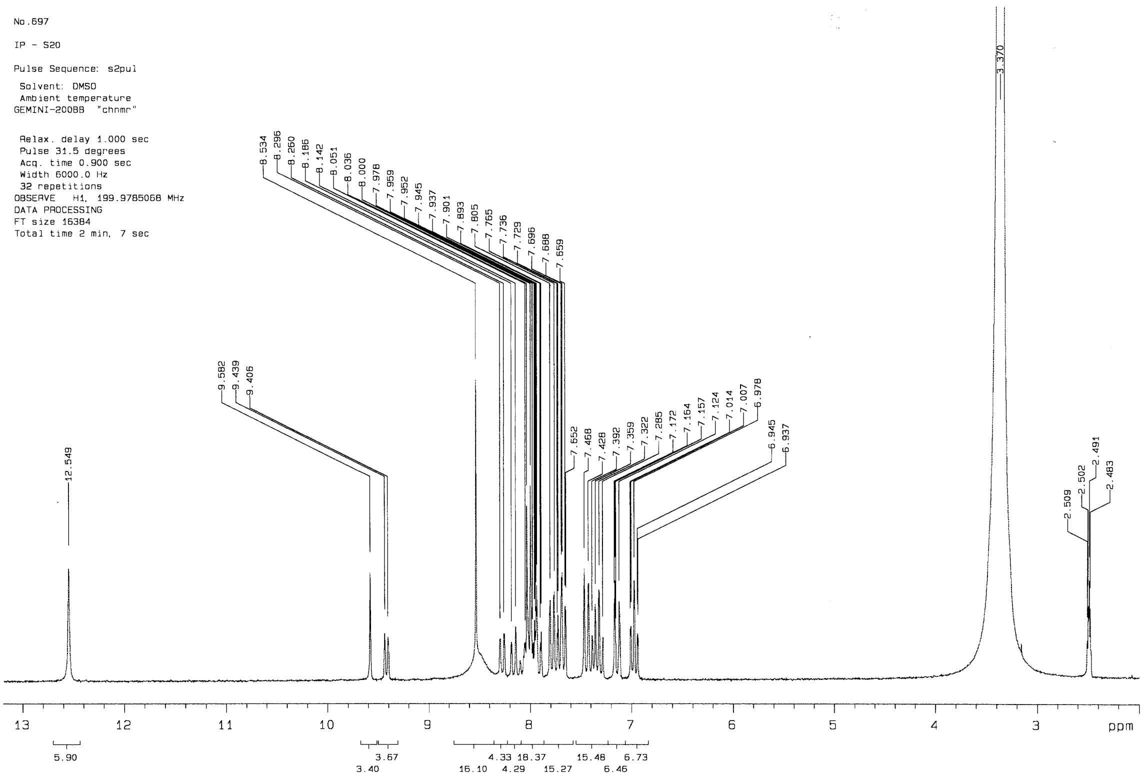

^{13}C spectrum of **DQ1**

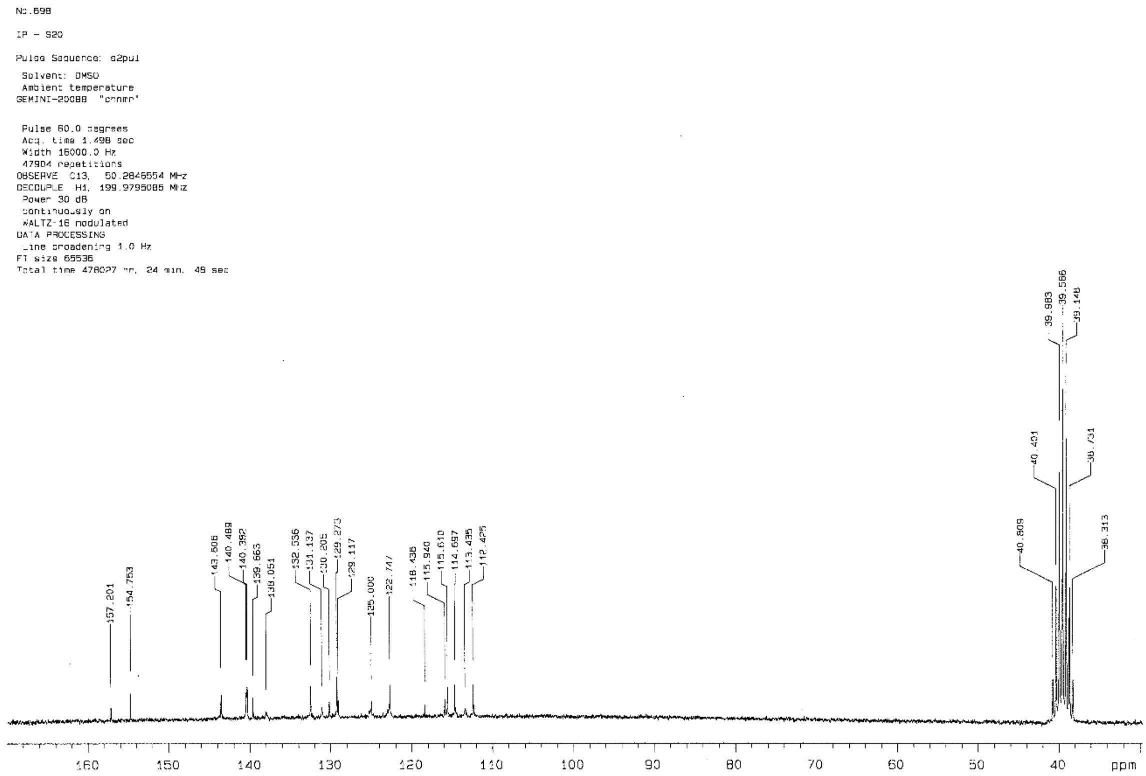

^{1}H spectrum of **DQ2**

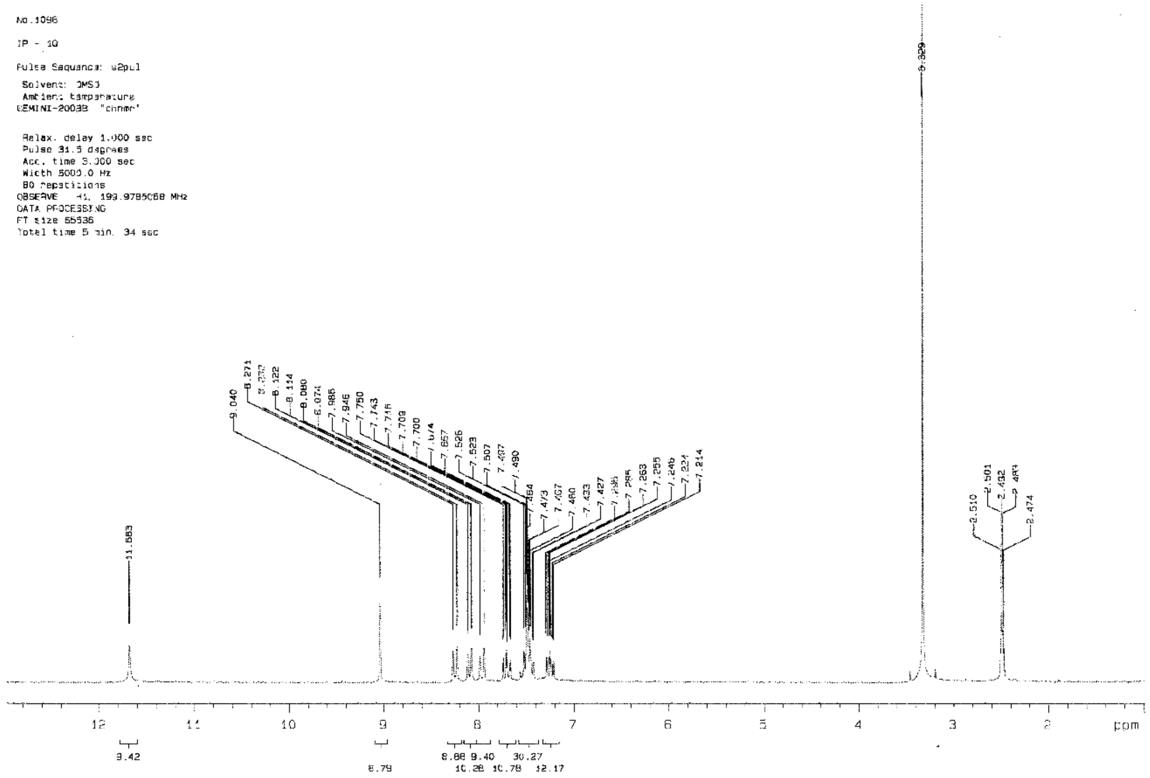

^{13}C spectrum of **DQ2**

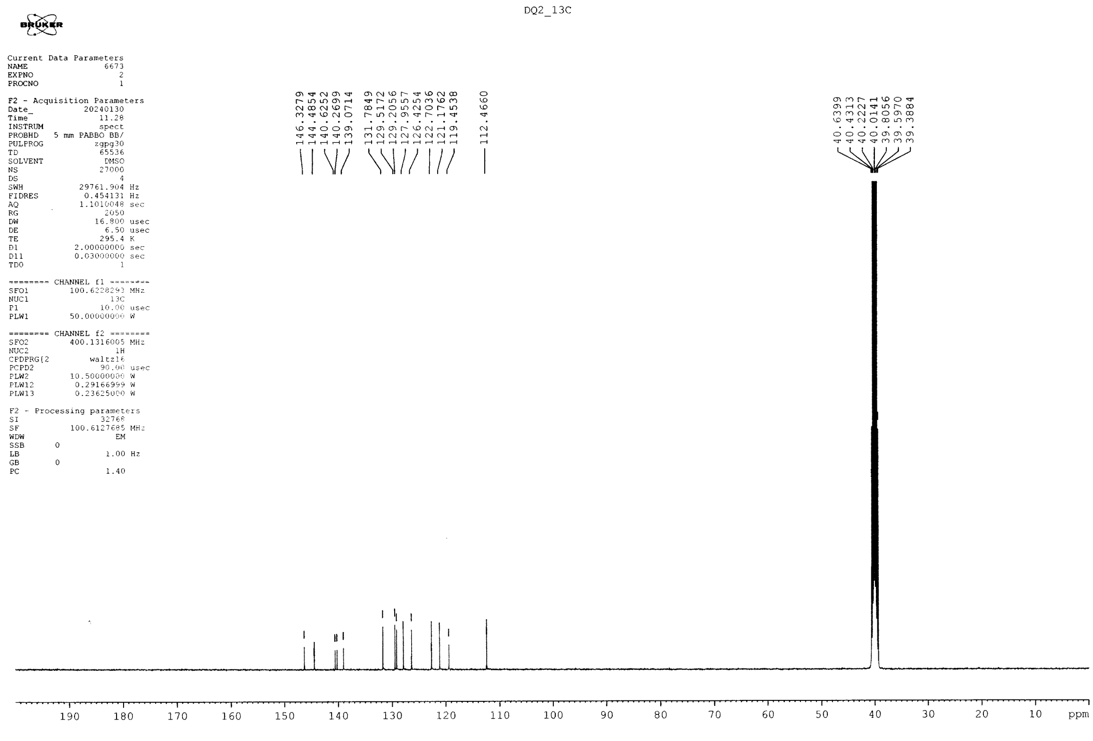

Enlarged spectrum in the range of 110-150 ppm.

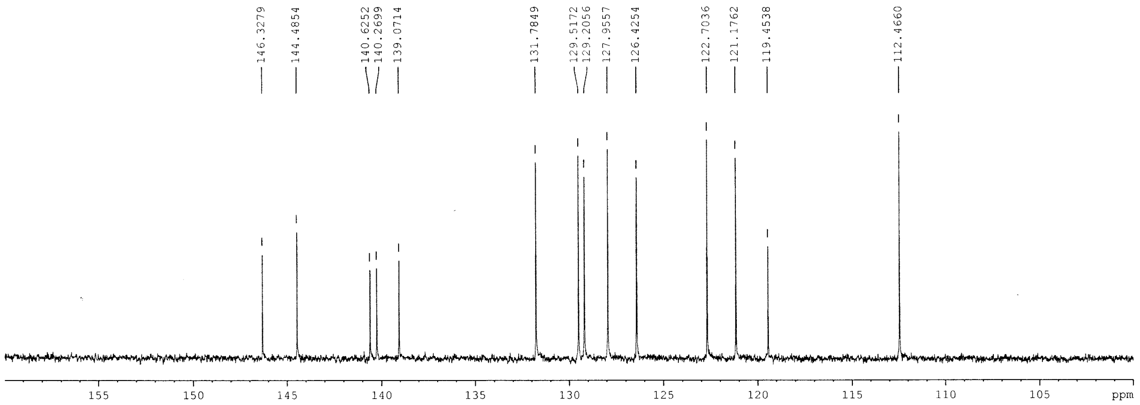

^{1}H spectrum of **DQ3**

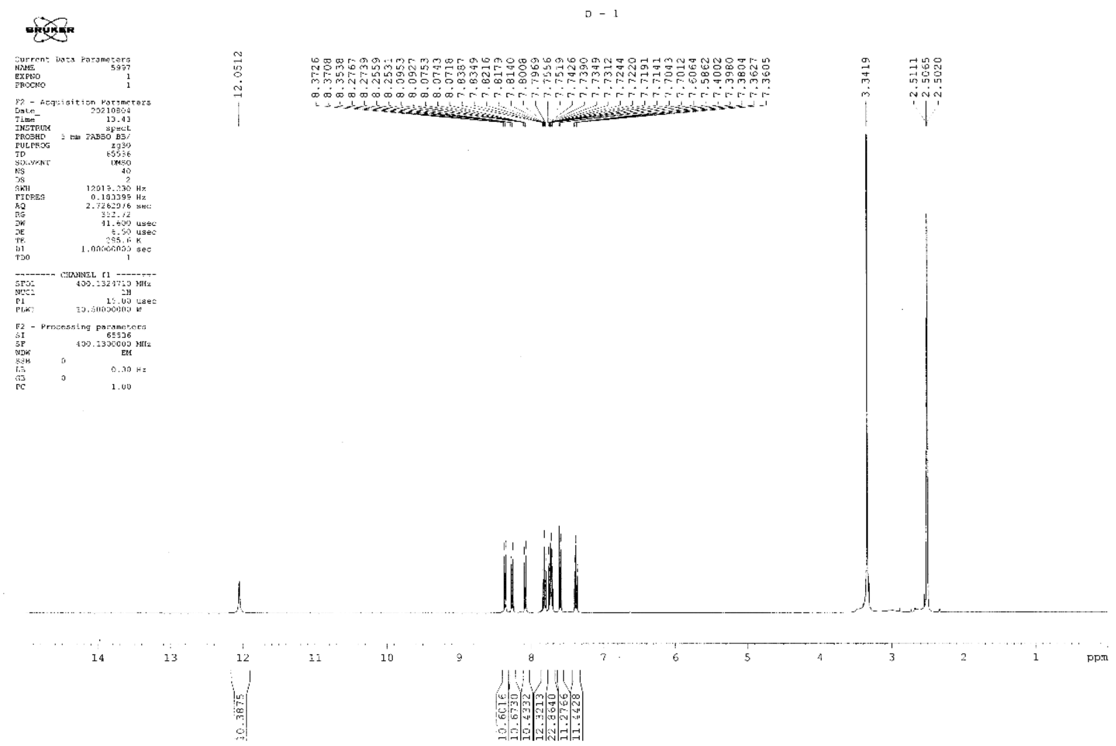

Enlarged spectrum in the range of 7-9.

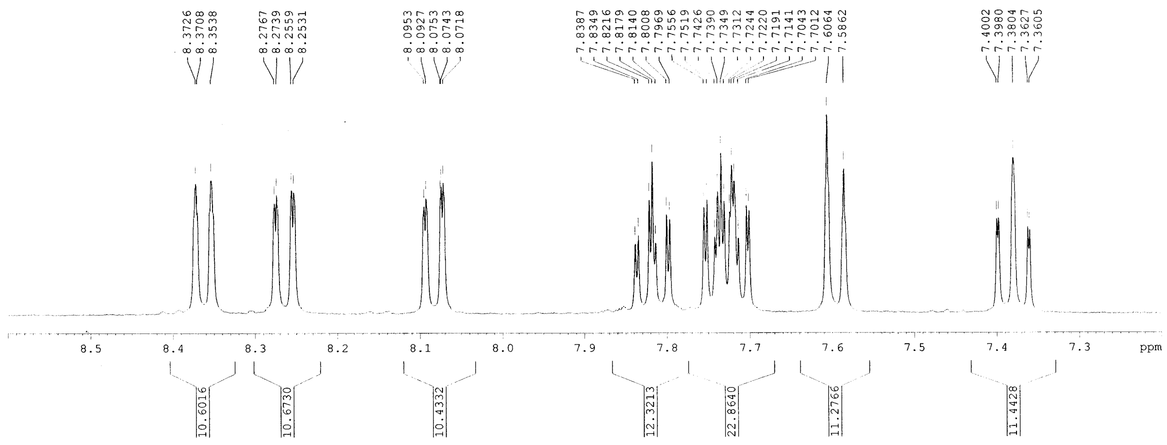

^{13}C spectrum of **DQ3**

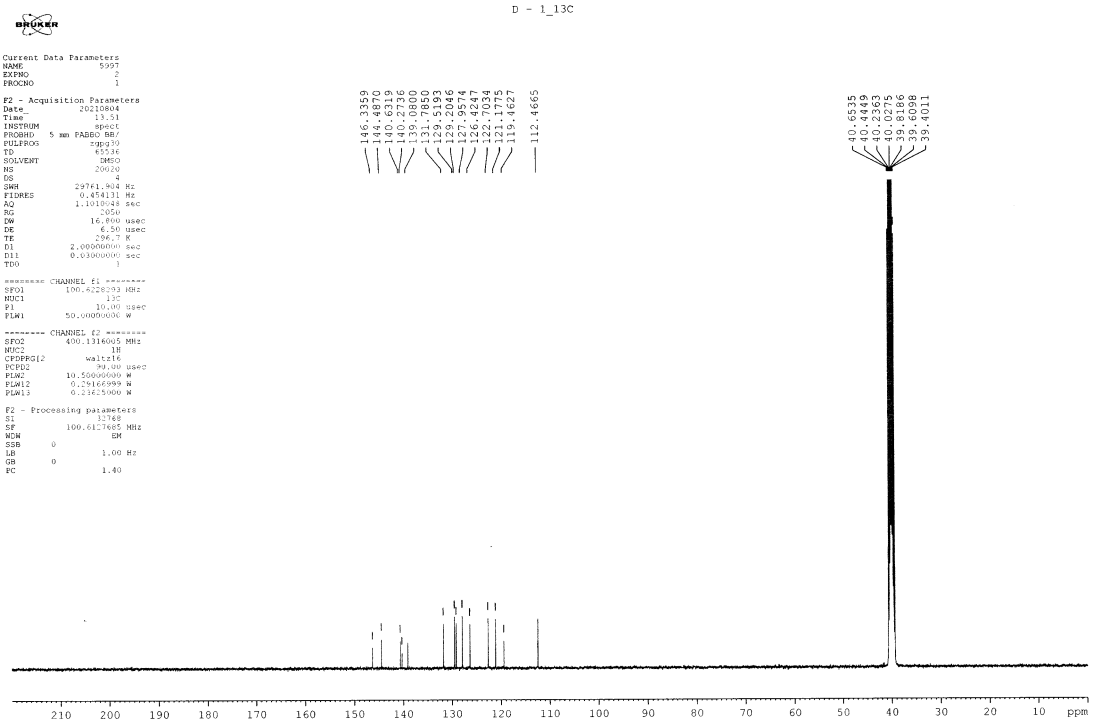

Enlarged spectrum in the range of 110-150 ppm.

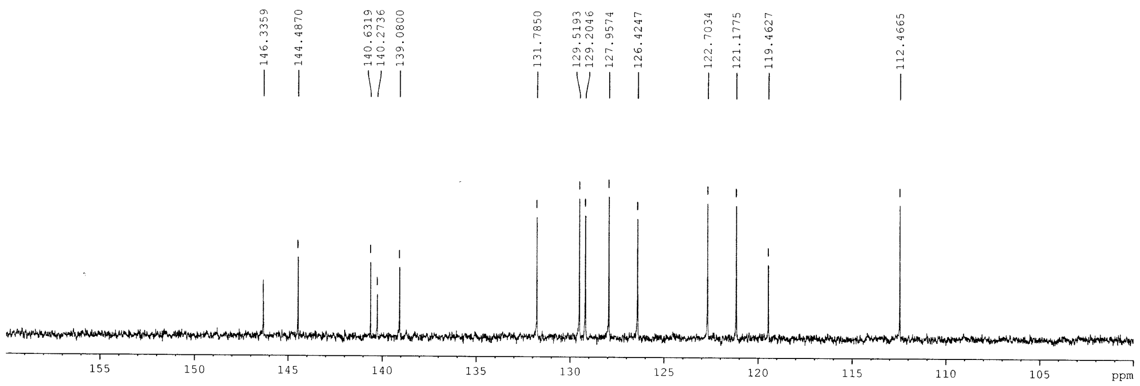

^{1}H spectrum of **DQ4**

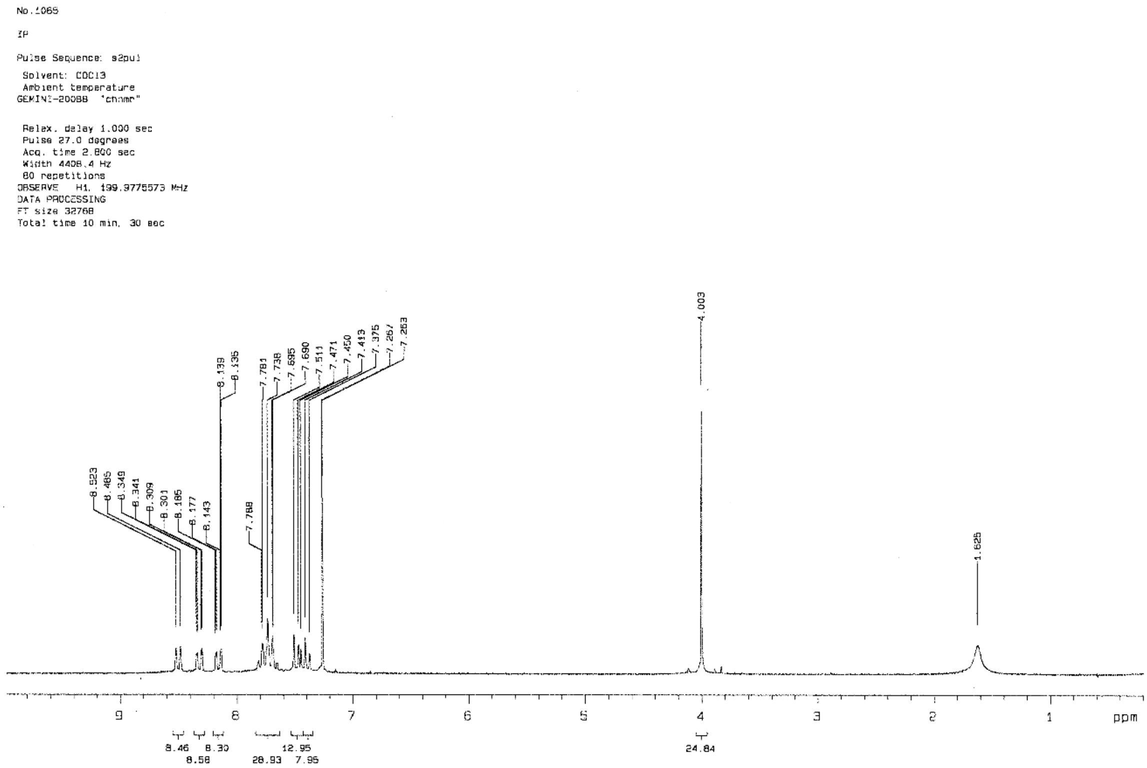

^{13}C spectrum of **DQ4**

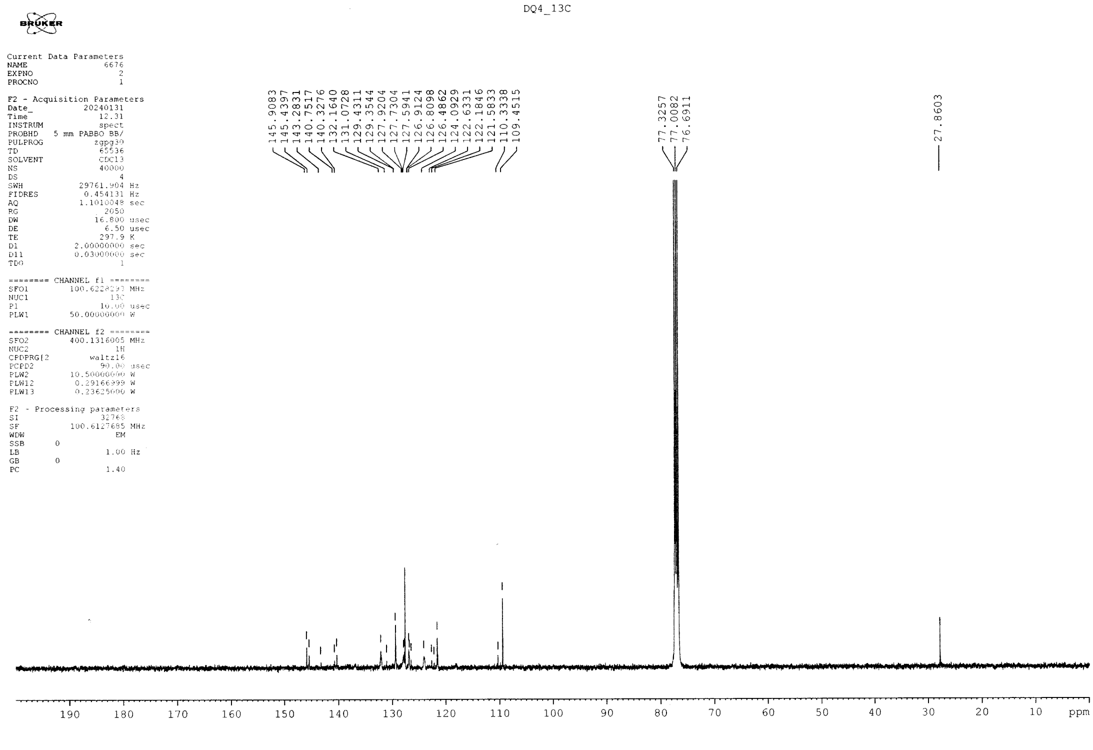

Enlarged spectrum in the range of 10-150 ppm.

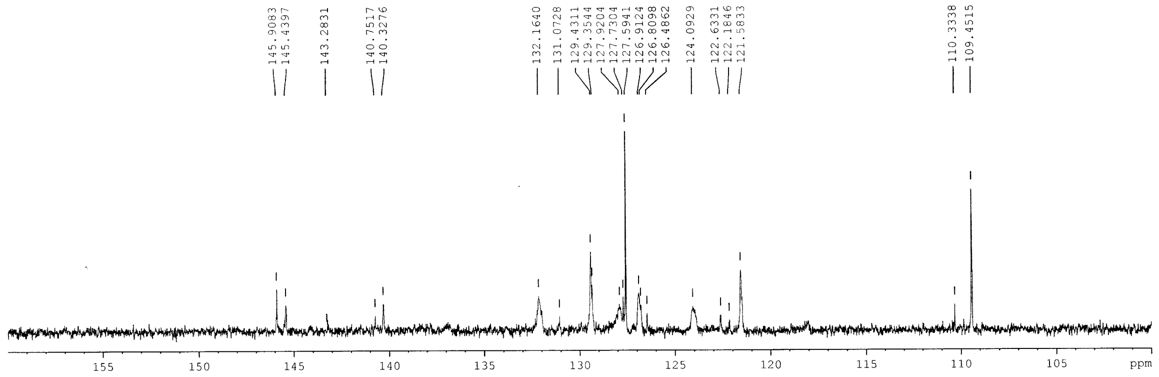

^{1}H spectrum of **DQ5**

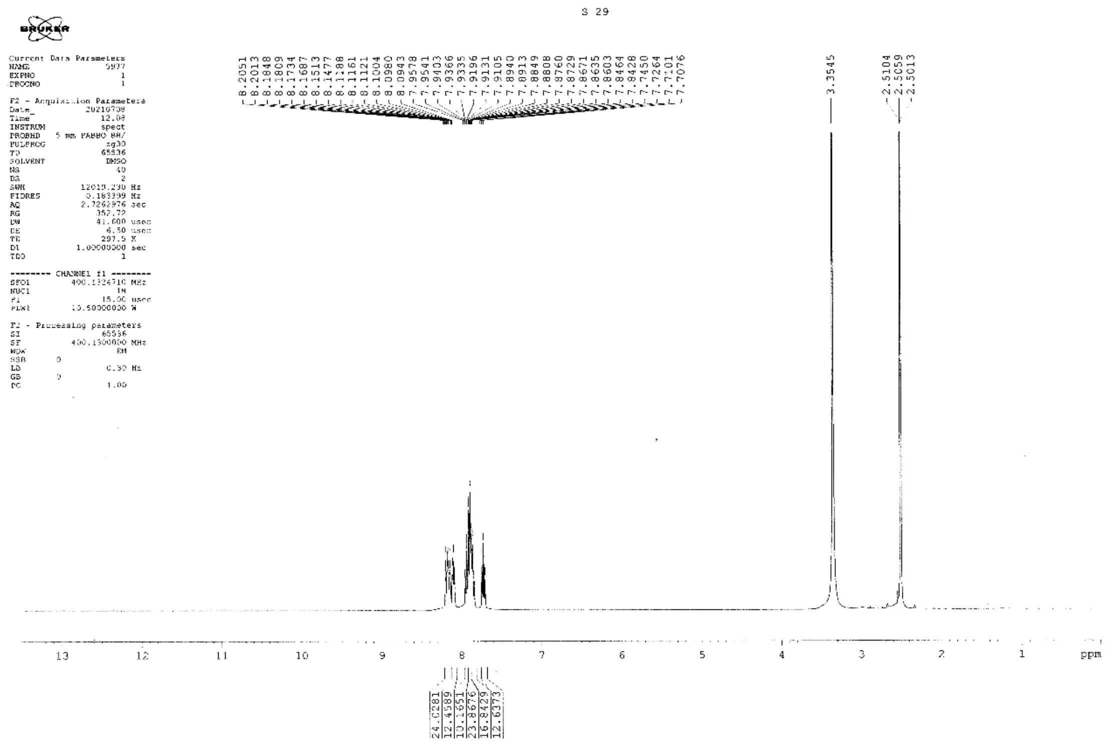

Enlarged spectrum in the range of 7-9.

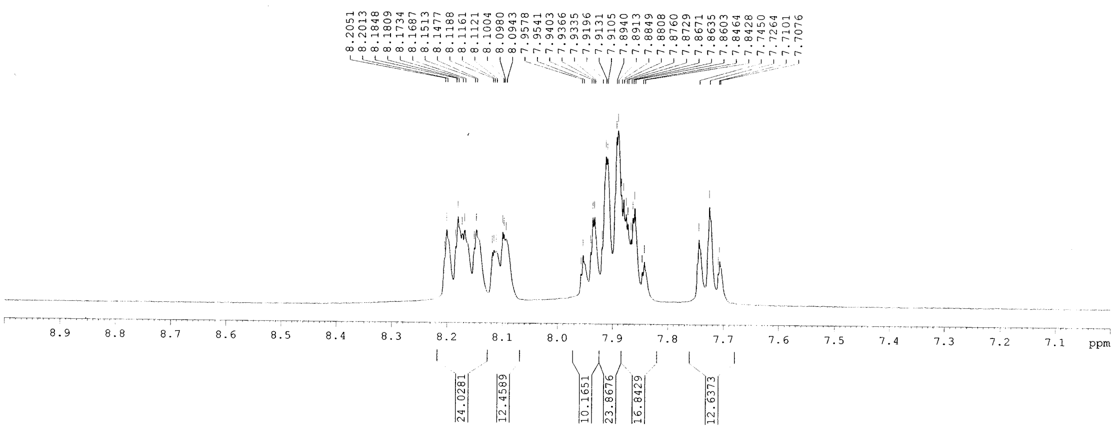

^{13}C spectrum of **DQ5**

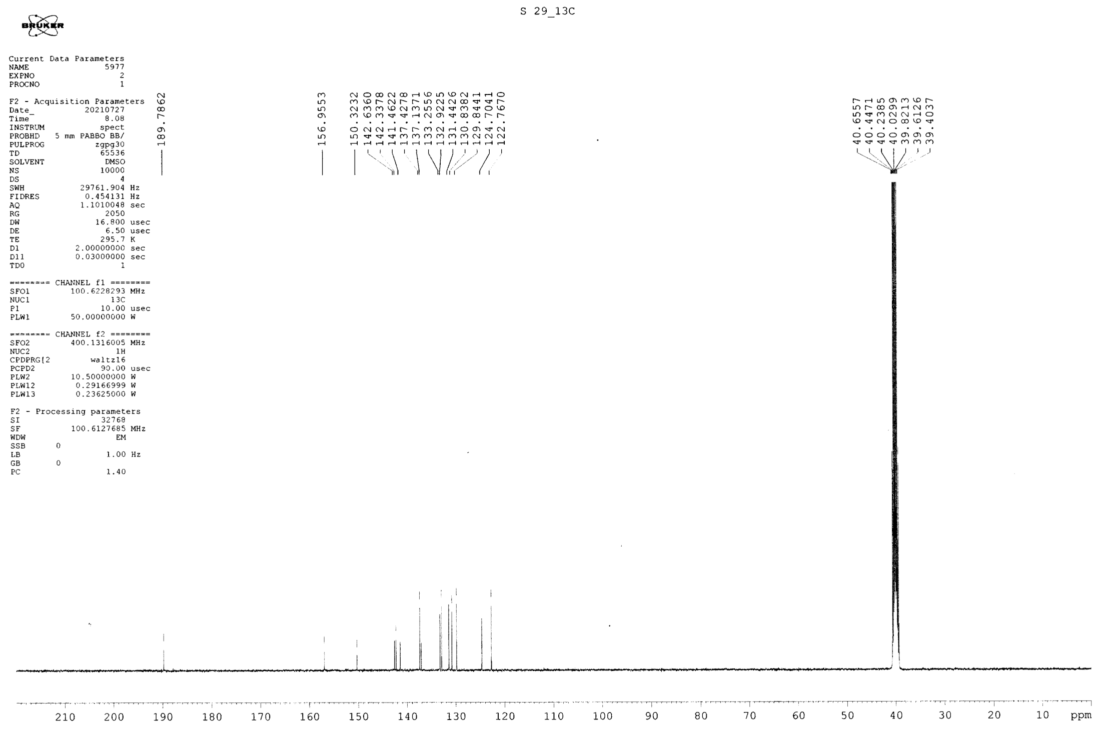

Enlarged spectrum in the range of 110-180 ppm.

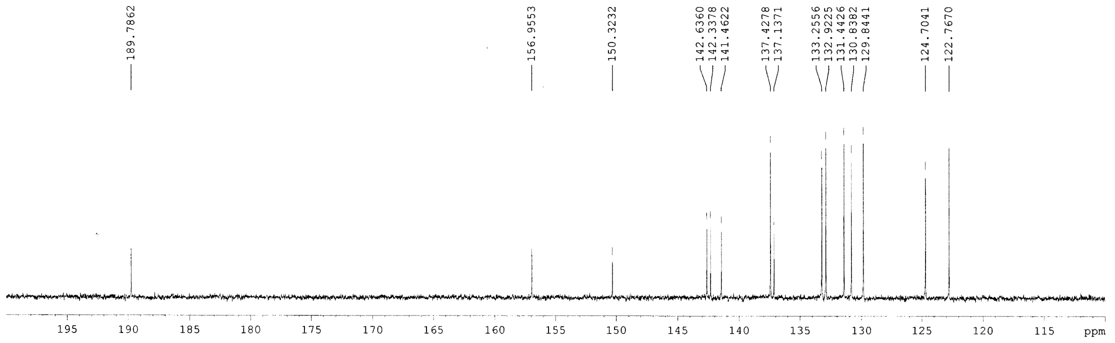

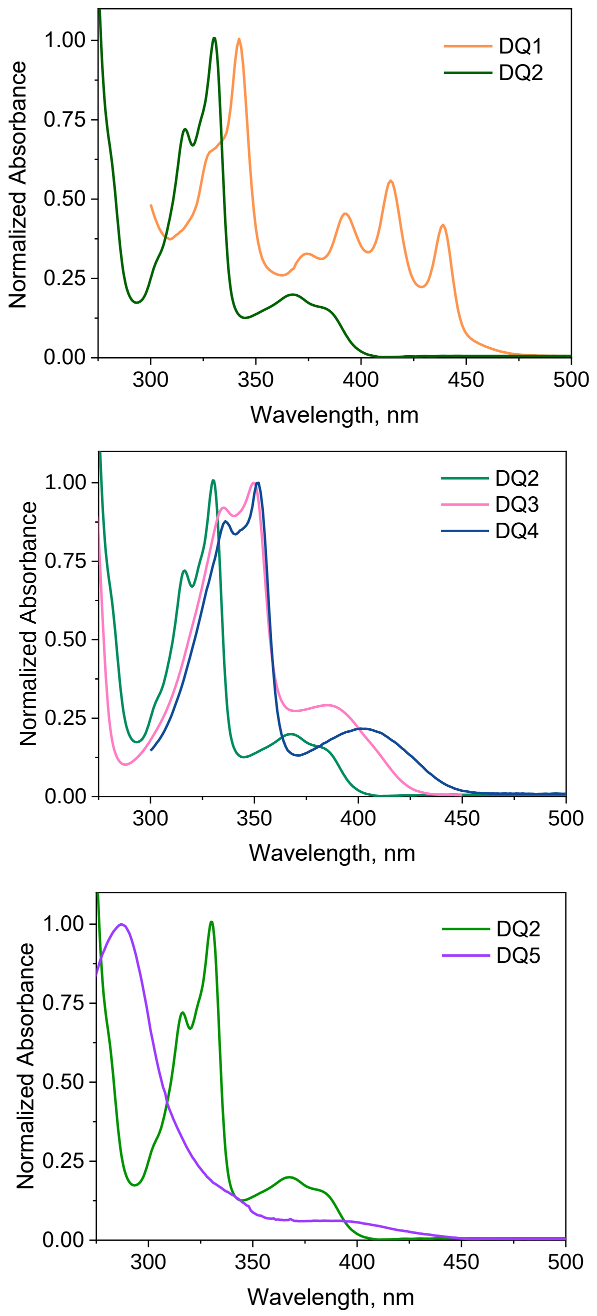

Figure S1. Normalized electronic absorption spectra of synthesized dyes in ethyl acetate.

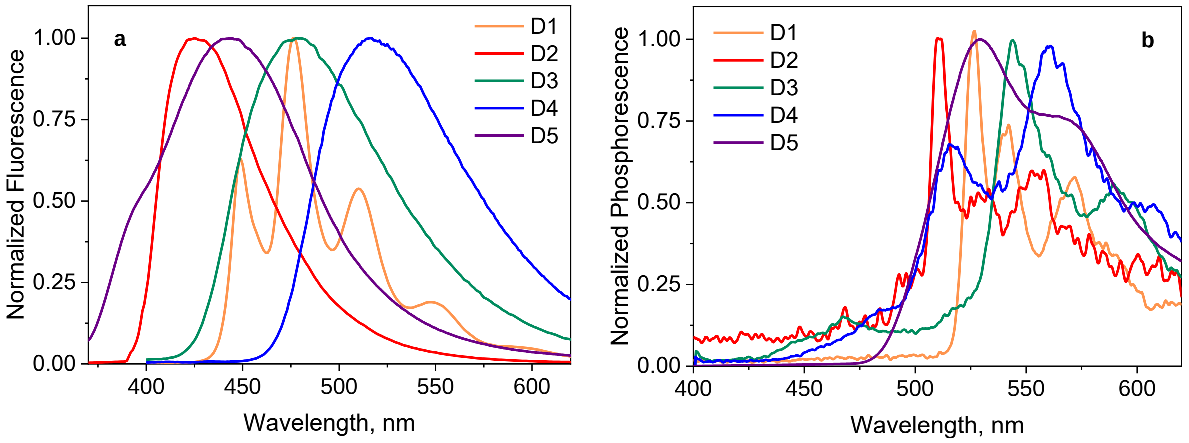

Figure S2. Normalized (a) fluorescence and (b) phosphorescence spectra of the synthesized dyes in 2-methyltetrahydrofuran at room temperature and at liquid nitrogen temperature, respectively.

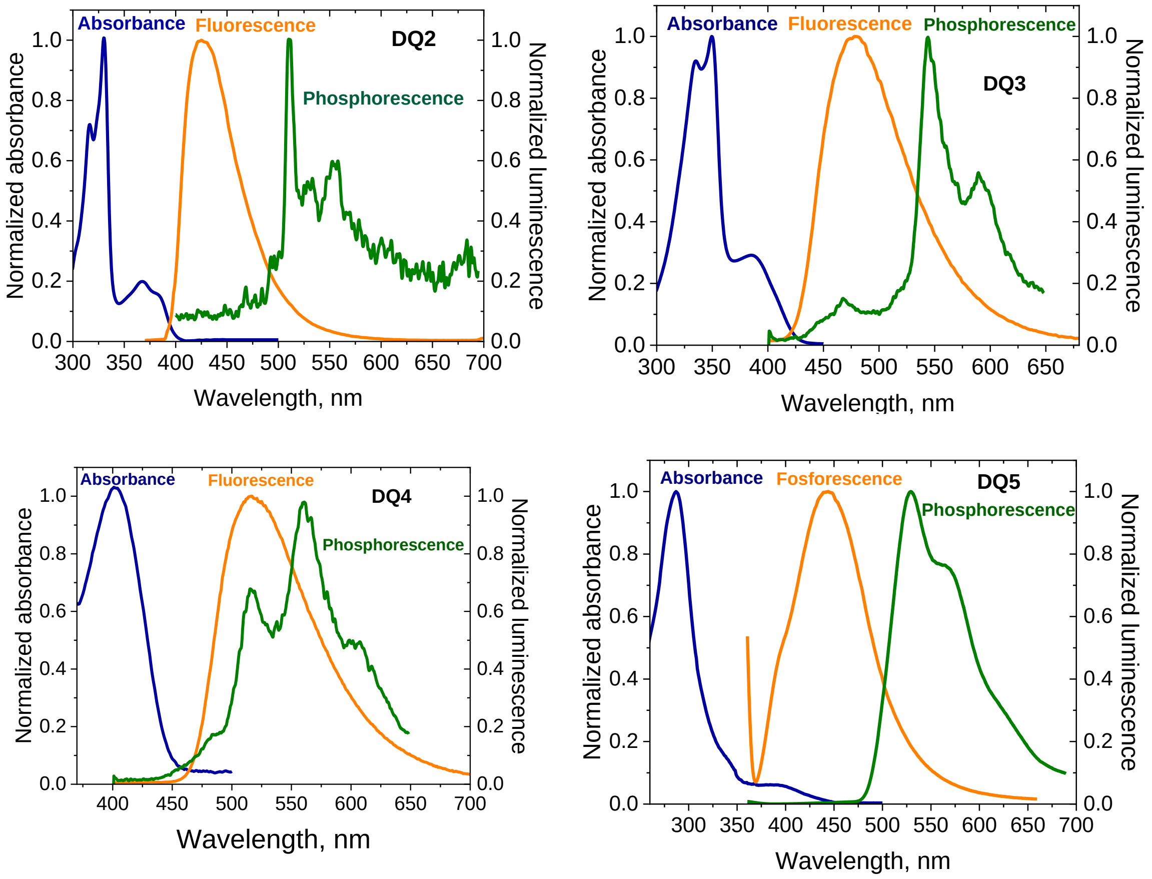

Figure S3. Electronic absorption spectrum, fluorescence and phosphorescence spectra of the tested dyes in 2-methyltetrahydrofuran.

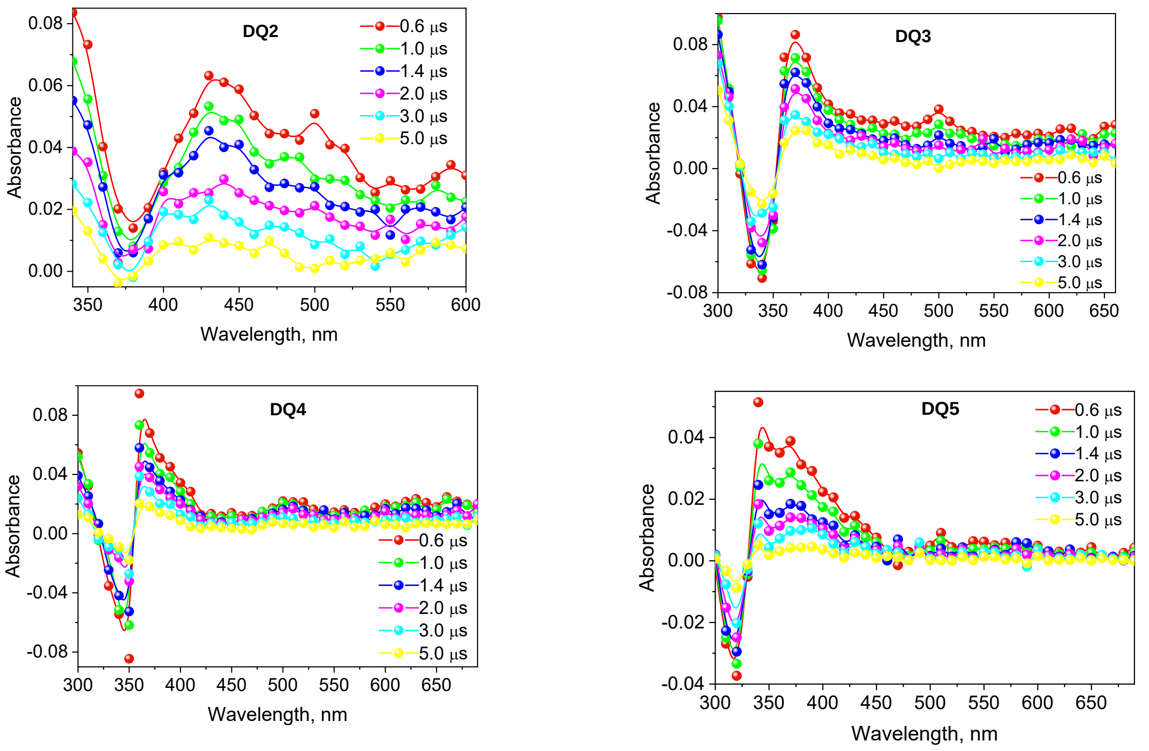

Figure S4. Absorption spectrum of the triplet state of the tested dyes recorded in deoxygenated acetonitrile.

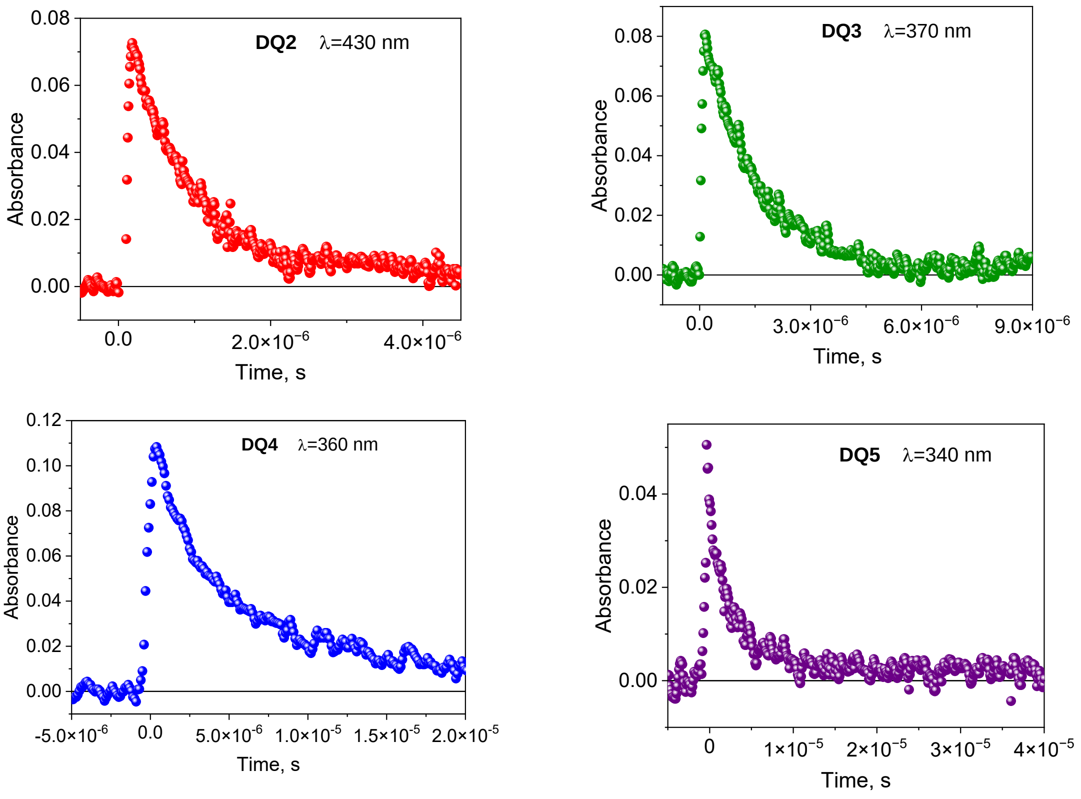

Figure S5. Kinetic curves of the disappearance of the triplet state recorded for the tested dyes at DQ2 - 430 nm, DQ3 - 370 nm, DQ4 - 360 nm, DQ5 - 340 nm.
